# Antimicrobial resistance profiles of *salmonella* spp. and *escherichia coli* isolated from fresh nile tilapia (*oreochromis niloticus*) fish marketed for human consumption

**DOI:** 10.1186/s12866-023-03049-8

**Published:** 2023-10-26

**Authors:** Millicent T. Mumbo, Evans N. Nyaboga, Johnson K. Kinyua, Edward K. Muge, Scholastica G. K. Mathenge, Henry Rotich, Geoffrey Muriira, Bernard Njiraini, Joshua M. Njiru

**Affiliations:** 1 Department of Biochemistry, University of Nairobi, Nairobi, Kenya; 2 Department of Biochemistry, Jomo Kenyatta University of Agriculture and Technology (JKUAT), Nairobi, Kenya; 3 Department of Medical Laboratory Science, Kenyatta University, Nairobi, Kenya; 4 Research and development Department, Kenya Bureau of Standards, Nairobi, Kenya

**Keywords:** Antimicrobial resistance, *Escherichia coli*, Health risk, Multi-drug resistant, *Oreochromis niloticus*, *S. typhimurium*

## Abstract

**Background:**

*Salmonella* spp. and pathogenic strains of *Escherichia coli* are among the major foodborne zoonotic pathogens. These bacterial pathogens cause human illnesses characterized by hemorrhagic colitis, vomiting, nausea, and other agent-related symptoms. The increasing occurrence of antimicrobial resistance in these pathogens is also a serious public health concern globally. Regular surveillance of phenotypes and genotypes of *Salmonella* spp. and *Escherichia coli* from animal-derived foods is necessary for effective reduction and control of these foodborne pathogens. This study was conducted to assess the occurrence, antimicrobial resistance, virulence genes and genetic diversity of *Salmonella* spp. and *E. coli* isolates from fresh Nile tilapia obtained from retail markets in Nairobi, Kenya.

**Methods:**

A total of 68 fresh Nile tilapia fish samples were collected from retail markets and used for isolation of *Salmonella* spp. and *E. coli*. Antimicrobial susceptibilities of the isolates weretested by Kirby-Bauer agar disc diffusion method. According to the antimicrobial resistance profiles, the multi-drug resistant isolates were identified by 16 S rRNA sequencing and phylogenetic analysis using the Bayesian inference method. The MDR *Salmonella* spp. and *E. coli* isolates were subjected to PCR-based screening for the detection virulence and antibiotic resistance genes.

**Results:**

The prevalence of contamination of the fish samples with *Salmonella* spp. and *E.coli* was 26.47% and 35.29% respectively. Overall phenotypic resistance among the *Salmonella* spp. ranged from 5.5% for ceftazidime, chloramphenicol, meropenem, nitrofurantoin and streptomycin and 22.2% for penicillin-G. For *E. coli* phenotypic resistance ranged from 4.2% for ceftazidime and chloramphenicol and 25% for rifampicin. Multi-drug resistance was observed in three *Salmonella* spp. and two *E. coli* isolates. Results of 16 S rRNA sequences, sequence alignment and phylogenic trees confirmed the identified MDR isolates as *S. typhymurium* WES-09, *S. typhymurium* MAK-22, *S. typhimurium* EMB-32 and *E. coli* MAK-26 and *E. coli* LAN-35. The presence of antibiotic-resistance genes belonging to β-lactamases, tetracycline, sulfonamide, trimethoprim and aminoglycosides-resistant genes were detected in all the identified MDR isolates.

**Conclusions:**

The findings from this study indicate that Nile tilapia (*Oreochromis niloticus*) sold in retail markets can acts as reservoirs of *Salmonella* spp. and *E*. *coli* pathogens linked to human disease, some of which were multidrug resistance to critically important antimicrobials. Both microorganisms are of zoonotic significance and represent a significant public health risk to the society.

**Supplementary Information:**

The online version contains supplementary material available at 10.1186/s12866-023-03049-8.

## Introduction

The rapid emergence of antimicrobial resistance (AMR) in the global ecosystem has become a threat to human, animal and environmental health [1–3]. Human, animal and environmental reservoirs contribute to AMR. Aquaculture production has been identified as a hot spot for the development of AMR, and transfer of drug-resistant microorganisms between food producing animals and humans [4, 5]. In particular, fish are reservoirs of zoonotic disease, infecting both the host and humans through food-borne disease or direct contact at the aquaculture facility [6].

Aquaculture sector is one of the fastest-growing sectors in the food industry, providing fish for human consumption as a source of protein and fatty acids [7]. Nile tilapia (*Oreochromis niloticus* Linnaeus, 1958) is the most popular fish in Kenya due to its palatability and economic price and as a resultthere has been rapid expansion of aquaculture by most farmers [8, 9]. In addition, the demand for Nile tilapia in local and international markets has intensified its production by farmers in Kenya. Nile tilapia was first cultured in Kenya in 1924 to boost the livelihoods of communities and to improve nutrition [10, 11] and is currently the most cultured fish speciesrepresenting about 90% of the national aquaculture production [12]. However, contamination is one of the main challenging factors either in the ponds or during harvesting or marketing, which can be a source of pathogens and may be a potential source of infection to humans [13]. Contaminated fish are unsuitable for human consumption since they can be a source of pathogenic bacteria.

Despite the high nutritional quality that links fish consumption to positive health effects in humans, the unsanitary conditions at fish farms and the occurrence of superbug bacteria in fish products have been reported as worrisome observations. This could pose a threat to human health, especially at these times when the demand of Nile tilapia as a source of animal protein in Kenya seems to be on the increase [12]. Aquatic ecosystems are vulnerable to many contaminants such as chemicals and drug residues as they are the recipients of run-offs from agricultural or livestock farms and healthcare facilities [14, 15]. Intensive fish farming has increased the uncontrolled use of antibiotics in the treatment of infections and as growth promoters resulting in the emergence of resistant bacteria strains [16]. This is of importance to human health as fish and fish products may be an important vehicle for the dissemination antibiotic-resistant pathogenic bacteria to other bacteria or directly to humans. Fish and fish products contaminated with human pathogens have been reported in many countries with *Salmonella* spp., *E. coli*, *Staphylococcus* species, *Vibrio* species, and *Pseudomonas aeruginosa* being the most important pathogens [17, 18].

*Salmonella* spp. and *E*. *coli* are the most important zoonotic bacterial pathogens that cause foodborne illnesses worldwide, and deaths due to the consumption of contaminated animal products [19]. Fish and fish products contaminated with human pathogens have been reported in many countries, with *Salmonella* spp. and *E*. *coli*, being the most common pathogens [17]. *Salmonella* spp. is not part of the healthy fish microbiota and its presence indicates fecal contamination either from polluted water or cross contamination during the production chain (unhygienic fish handling, fish processing or marketing) [20, 21]. Pathogenic *E*. *coli* can cause severe foodborne disease and is a pathogen of concern because of the prospective challenges of treatment when humans are infected. Studies targeting the contamination of fish with *Salmonella* spp. and *E. coli* are therefore important as there is limited information particularly in the study area, despite the increased popularity of fish consumption.

Studies have reported the presence of antibiotic-resistant *Salmonella* spp. and *E. coli* in fish and fish products [18, 22, 23]. Antibiotic resistance is growing and has affected critically important classes of bactericidal antibiotics used to treat bacterial infections in humans [24]. One of the most important resistance mechanisms in Gram-negative bacteria against beta-lactam antibiotics is induced by the production of beta-lactamases [24]. Extended β-lactamases (ESBL) are plasmid-mediated β–lactamase enzyme recognized for their remarkable ability to hydrolyze penicillin, 3^rd^ and 4^th^ generation cephalosporins and monobactams except for carbapenem and cephamycin [25]. These enzymes emerged from ^*bla*^TEM-1, ^*bla*^TEM-2 and ^*bla*^SHV as narrow-spectrum parents. Recently, ^*bla*^CTX-M, a new class of ESBL genes, appeared to have gained global attention. The rates of CTX-M producing bacteria have increased worldwide and the situation is more complicated as these enzymes confer co-resistance to other commonly used antibiotic classes [26–28]. In aquaculture, a variety of these antibiotics are authorized for use, resulting in the emergence of ESBL-producing Gram-negative bacteria.

The occurrence and increase of bacterial strains resistant to routinely used antibiotics in fish hatcheries and their possible human health implications is calling for intensified surveillance systems. There is limited scientific data on the antimicrobial resistance of food pathogens in fish from retail markets in Kenya. Therefore, monitoring the prevalence of antibiotic resistance microorganisms is necessary to provide knowledge about the magnitude of the problem and help government authorities to evaluate the effectiveness of the control measures. In this study, we hypothesized that the *Salmonella* spp. and *E. coli* contaminants isolated from Nile tilapia marketed in the Nairobi region of Kenya are highly resistant to multiple antibiotics. The objective of this study was to establish the occurrence, virulence potential and antimicrobial resistance of *Salmonella* spp. and *E. coli* isolated from fresh Nile tilapia fish from retail markets. Additionally, the genetic diversity among the multi-drug resistant *Salmonella* spp. and *E. coli* isolates from Nile tilapia from retail markets was determined. Furthermore, for every MDR *Salmonella* spp. and *E. coli* isolate, ESBL production and ESBL gene presence were determined by double-disc synergy test and polymerase chain reaction (PCR) tests, respectively.

## Materials and methods

### Study site and fish sample collection

The study was conducted in five sub-Counties (Kasarani, Langata, Westlands, Embakasi and Makadara) under the administration of Nairobi County, Kenya. The five sub-counties and their locations were Kasarani (latitude − 1.227841 and longitude 36.905729), Makadara (latitude − 1.296140 and longitude 36.871042), Westlands (latitude − 1.2683 and longitude 36.8111), Embakasi (latitude − 1.3000 and longitude 36.9167) and Lang’ata (latitude − 1.366667 and longitude 36.733333). This was a cross section study in which a total of 68 fish samples were collected retail markets of the five sub-counties in Nairobi, Kenya. The samples collected from each of the sub-Counties were 14 each for Kasarani, Makadara, Westlands sub-Counties and 13 each for Embakasi and Langata. The number of fish collected in every market depended on the availability of fish vendors. The fish samples were collected in sterile zip-lock polypropylene bags and transported in cool boxes with ice packs to the Microbiology section at the Department of Biochemistry, University of Nairobi.

### Isolation and identification of presumptive *Salmonella* spp. and *Escherichia coli* on selective and differential media

The media used were xylose lysine deoxychocolate agar (HIMedia Laboratories Pvt. Mumbai, India), triple sugar iron (TSI) agar (HIMedia Laboratories Pvt. Mumbai, India) (for *Salmonella* spp.), brain-heart infusion (BHI) agar (HIMedia Laboratories Pvt. Mumbai, India) (for both *Salmonella* spp. and *E. coli*), and MacConkey Agar (HIMedia Laboratories Pvt. Mumbai, India) and Eosin Methylene Blue (EMB) agar (HIMedia Laboratories Pvt. Mumbai, India) (for *E. coli*). Fish were aseptically dissected to obtain 15 g of the sample (the gills and flesh of fish) which were added to 50 ml buffered peptone water (HIMedia Laboratories Pvt. Mumbai, India) and homogenized using a stomacher 400 circulator (Seward Ltd, England). Five milliliters of each tissue homogenate was analyzed for any enterobacteriaceae. The homogenate was inoculated on Brain-heart infusion agar. The inoculated plates were incubated at 37 °C for 24 h. Sub-culturing was done on MacConkey, Eosin Methylene Blue (EMB) and Xylose Lysine Deoxychocolate agar plates to obtain pure cultures of the respective bacteria isolates [29]. The bacterial isolates were confirmed by standard morphological characteristics (shape, size, surface texture, margins and elevation, colour and opacity) and biochemical tests as described by Cheesbrough [29]. The biochemical tests performed were triple sugar iron test (TSI), methyl red test (MR), Voges-Proskauer test (VP), citrate utilization test (CIT), urease test (URE), catalase test (CAT), oxidase test (OX) and hydrogen sulphide (H_2_S) production test.

### Enumeration of total bacterial load

The homogenized samples were serially diluted (10^− 1^ to 10^− 3^) and cultured onto their respective media [30]. The plates were inverted and incubated at 37 °C for 18 to 24 h [29]. The bacterial load was determined by the counting the number of discrete colonies using the viable plate count method [31].

### Antimicrobial drug susceptibility testing

Antimicrobial drug susceptibility testing of *Salmonella* spp. and *E.coli* isolates to antibiotic discs (HiMedia, India) of penicillin (10 µg), vancomycin (30 µg), rifampicin (5 µg), ampicillin (10 µg), cefepime (30 µg), cefpodoxime (10 µg), chloramphenicol (30 µg), nitrofurantoin (300 µg), ceftazidime (30 µg), meropenem (10 µg), and streptomycin (10 µg) was determined on Mueller Hinton agar plates using the Kirby–Bauer agar disc diffusion method [32]. These antimicrobials were selected based on the availability and upon the recommendation of World Health Organization and World Organization for Animal Health (OIE) for use in both human and food-producing animals [33, 34]. The test organism was uniformly seeded over the Muller Hinton Agar (MHA) (Oxoid Basingstoke, England) surface and exposed to the concentration of the antibiotic and incubated at 37 °C for 24 h. Inhibition zone diametersaround the discs were measured to the nearest millimeters and classified as resistant, intermediate or susceptible as per the criteria of Clinical Laboratory Standards Institute 2021 [35]. *Salmonella* spp. and *E. coli* isolates resistant to antibiotics from three or more antimicrobial classes were defined as multi-drug resistant (MDR) isolates.

### Determination of multiple antibiotic resistance (MAR)

Multiple antibiotic resistance (MAR) index was analyzed as described by Krumperman [36]. MAR index was calculated by dividing the number of antibiotics to which the test isolate depicted resistance to the number of antibiotics to which the test isolate was evaluated for susceptibility. Multiple antibiotic-resistant phenotypes (MARPs) for each sampling site were generated for isolates that showed resistance to more than three antibiotics following the method described by Wose et al. [37]. The antibiotic resistance pattern, number of antibiotics to which the isolates were resistant, frequencies and percentages were obtained from Kirby Bauer tests.

### Phenotypic detection of ESBL production

Phenotypic testing of ESBL production was tested by the Modified Double Disc Synergy Test (DDST) [38] by using a disc of amoxicillin with clavulanate (20/10 µg) along with cefotaxime (30 µg) and ceftazidime (30 µg). A standard inoculum (0.5 McF) of the *Salmonella* spp. and *E. coli* isolates were swapped on the surface of Mueller-Hinton II (MH II) agar plates (Biolife, IT). An amoxicillin with clavulanate (20/10 µg) disc was placed at the center of MH II agar plates while discs of cefotaxime (30 µg) and ceftazidime (30 µg) were placed in close proximity of 15 mm distance. Any distortion or increase in the inhibition zone of cephalosporin antibiotics towards the disc of amoxicillin-clavulanate was considered as positive for the ESBL production.

### Molecular identification and phylogenetic analysis of multidrug resistant (MDR) bacteria

The 16S ribosomal RNA (16S rRNA) PCR amplification and sequencing of the amplicons was used in the identification of MDR *Salmonella* spp. and *E. coli* isolates. Multidrug resistant bacteria were sub-cultured by picking a single colony followed by genomic DNA extraction (Qiagen kit, Hilden, Germany), as per manufacturer’s instructions. The DNA was used for polymerase chain reaction (PCR) using primers targeting the 16S rRNA gene (Table 1). The PCR mixture consisted of 25 µl GoTaq Green MasterMix (Promega), 2 µl DNA, 1 µl of each forward and reverse primer, and nuclease-free water up to 50 µl. Recycling conditions and time of the primers during PCR are shown in Table 1. The PCR products were resolved by 1.5% (w/v) agarose gel electrophoresis (Qiagen, Hombrechtikon, Switzerland). The Agarose gels stained with gelred were viewed under a Gel imager (Biorad Gel Doc XR System, USA). QIA-quick kit (Qiagen, Hilden, Germany) was used to purify PCR products (amplicons) according to the manufacturer’s instructions. Sanger method (Dideoxy sequencing of DNA) was used to obtain the nucleotide sequences of the amplicons (Macrogen, Europe).

**Table 1:** 16S rRNA, virulence and antibiotic resistance genes primer sequences, expected amplicons sizes and PCR cycling conditions

Target microorganism	Target gene	Primer sequence (5′ → 3′)	Amplicon size (bp)	PCR cycling condition	Reference
*Salmonella* spp.*/ E. coli*	16S rRNA	F: GAG TTT GAT CCT GGC TCA R: TAC GGC TAC CTT GTT ACG ACT T	1500	5 min initial denaturation at 94 °C followed by 35 cycles of 94 °C for 40 s, 58 °C for 40 s, 72 °C for 40 s and final extension at 72 °C for 7 min	[41]
*Salmonella* spp.	*InvA* (Virulence gene)	F: ACA GTG CTC GTT TAC GAC CTG AAT R: AGA CGA CTG GTA CTG ATC GAT AAT	244	5 min initial denaturation at 94 °C followed by 35 cycles of 94 °C for 1 min, 58 °C for 1 min, 72 °C for 1 min and final extension at 72 °C for 10 min	[42]
*Salmonella* spp.	*hilA* (Virulence gene)	F:CGTGAAGGGATTATCGCAGT R: TCCGGGAATACATCTGAGC	600	5 min initial denaturation at 94 °C followed by 35 cycles of 94 °C for 1 min, 65 °C for 1 min, 72 °C for 1 min and final extension at 72 °C for 10 min	[43]
*E. coli*	*uidA* (Virulence gene)	F: AAA ACG GCA AGAAAA AGC AG R: ACG CGT GGT TAACAG TCT TGC G	147	5 min initial denaturation at 94 °C followed by 35 cycles of 94 °C for 40 s, 58 °C for 40 s, 72 °C for 40 s and final extension at 72 °C for 7 min	[44]
**Antibiotic resistance genes**	^*bla*^TEM-1	F: TTG GGT GCA CGA GTGGGT R: TAA TTG TTG CCG GGA AGC	500	5 min initial denaturation at 94 °C followed by 35 cycles of 94 °C for 1 min, 57 °C for 1 min, 72 °C for 1 min and final extension at 72 °C for 10 min	[45]
	^*bla*^CMY-2	F: ATA ACC ACC CAG TCA CGC R: CAG TAG CGA GAC TGC GCA	600	5 min initial denaturation at 94 °C followed by 35 cycles of 94 °C for 1 min, 58 °C for 1 min, 72 °C for 1 min and final extension at 72 °C for 10 min	[45]
	^*bla*^CTX-M	F: CGC TTT GCG ATG TGC AG R: ACC GCG ATA TCG TTG GT	590	5 min initial denaturation at 94 °C followed by 35 cycles of 94 °C for 1 min, 52 °C for 1 min, 72 °C for 1 min and final extension at 72 °C for 10 min	[46]
	^*bla*^Z	F: ACT TCA ACA CCT GCT GCT TTC R: TGA CCA CTT TTA TCA GCA ACC	490	94 °C for 5 min followed by 30 cycles of denaturation 94 °C for 30 s, annealing 60 °C for 30 s, extension 72 °C for 90 s and final incubation at 72 °C for 5 min	[47]
	*catI*	F: AGTTGCTCAATGTACCTATAACC R: TTGTAATTCATTAAGCATTCTGCC	280	5 min at 94 °C, followed by 30 cycles of 94 °C for 30 s, 50 °C for 30 s and 72 °C for 1.5 min and final incubation at 72 °C for 5 min.	[48]
	*sul2*	F: CGG CAT CGT CAA CAT AAC C R: GTG TGC GGA TGA AGT CAG	720	5 min initial denaturation at 94 °C followed by 35 cycles of 94 °C for 1 min, 58 °C for 1 min, 72 °C for 1 min and final extension at 72 °C for 10 min	[49]
	*tetA*	F: GCT ACA TCC TGC TTG CCT TC R: CAT AGA TCG CCG TGA AGA GG	210	5 min initial denaturation at 94 °C followed by 35 cycles of 94 °C for 1 min, 60 °C for 1 min, 72 °C for 1 min and final extension at 72 °C for 10 min	[50]
	*tetC*	F: CTT GAG AGC CTT CAA CCC AG R: ATG GTC GTC ATC TAC CTG CC	418	5 min initial denaturation at 94 °C followed by 35 cycles of 94 °C for 1 min, 62 °C for 1 min, 72 °C for 1 min and final extension at 72 °C for 10 min	[50]
	*dfrA7*	F: GGT AAT GGC CCT GAT ATC CC R: TGT AGA TTT GAC CGC CAC C	280	5 min initial denaturation at 94 °C followed by 35 cycles of 94 °C for 1 min, 58 °C for 1 min, 72 °C for 1 min and final extension at 72 °C for 10 min	[51]
	*strA*	F: CTT GGT GAT AAC GGC AAT TC R: CCA ATC GCA GAT AGA AGG C	548	5 min initial denaturation at 94 °C followed by 35 cycles of 94 °C for 1 min, 56 °C for 1 min, 72 °C for 1 min and final extension at 72 °C for 10 min	[52]
	*aadA*	F: GTG GAT GGC GGC CTG AAG CC R: AAT GCC CAG TCG GCA GCG	525	5 min initial denaturation at 94 °C followed by 35 cycles of 94 °C for 40 s, 60 °C for 40 s, 72 °C for 40 s and final extension at 72 °C for 7 min	[52]

The obtained sequences were edited manually using BioEdit v7.0.5.3 [39] to remove gaps and minimize insertions and aligned in MUSCLE [40]. BLASTn searches were done using target sequences from the study isolates and compared with those obtained from the GenBank database. The homologous sequences to the queries were selected based on the Expectation value (E value) as well as query coverage and percent identity. Phylogenetic analysis was done using the Bayesian inference method by MrBayes software v3.2.7 (https://nbisweden.github.io/MrBayes/).The resulting phylogenetic trees constructed by MrBayes were visualized on FigTree software v1.4.4 (http://tree.bio.ed.ac.uk/software/figtree/).

### Molecular detection of virulence-associated and antibiotic resistance genes

Genes encoding resistance to antimicrobialswereidentifiedfrom the antibiotic resistant bacterial isolates. The PCR mixture consisted of 12.5 µl GoTaq Green MasterMix (Promega, United States), 1 µl DNA, 0.5 µl of eachof the forward and reverse primers, and nuclease-free water up to 25 µl.The primer sequences and PCR cycling conditions are shown in Table 1. The reaction was done using a Proflex PCR system (Applied Biosystems™, United States).The primers used were; *uidA*, *invA*, *hilA* for virulence genes (Table 1) and ^*bla*^TEM-1, ^*bla*^CMY-2, ^*bla*^CTX-M, ^*bla*^Z (β-lactamases-encoding genes), *catI* (chloramphenicol resistant gene), *tetA*, *tetC* (tetracycline resistant genes), *sul2* (sulfonamide-resistant genes), *dfrA7* (trimethoprim-resistant genes), *strA*, *aadA* (aminoglycosides resistant genes) for antibiotic resistance genes (Table 1).

The PCR products were resolved by 1.5% (w/v) agarose gel electrophoresis in 1× TAE buffer for 1 h at 100 V. Agarose gels stained with gelred were viewed under a gel imager (Biorad Gel Doc XR System, USA) and photographed.

### Statistical analysis

Statistical analysis was performed using Minitabv17.1 statistical software (Minitab, LLC). In order to compare bacterial counts in five sampling locations, chi-square (X^2^) was used. P-value of ≤ 0.05 was regarded significant. A Microsoft Excel spreadsheet software package was also used to generate graphs and charts.

## Results

### Phenotypic and biochemical characteristics of isolated bacteria

All the 42 recovered bacterial isolates had round colonies ranging from 2 to 4 mm in diameter and were opaque on their respective selective media. The colonies were round with entire margins while the texture was either firm or mucoid (Supplementary Table S1). *Salmonella* spp. colonies on xylose lysine deoxycholate (XLD) agar appeared red with black centres (Supplementary Fig. S1). *Salmonella* spp. on triple sugar iron (TSI) agar had a characteristic appearance of red slant surface, yellow butt, blackish growth on the media with gas production (cracks were observed in the media) (Supplementary Fig. S2). The colonies with black centres in XLD and blackish growth on TSI were considered as presumptive *Salmonella* spp. positive. *E. coli* colonies on MacConkey agar revealed the growth of bacteria after 24 h of incubation 37 °C and appeared bright pink colour colonies. Eosin Methylene Blue (EMB) agar streaked with *E*. *coli* isolates revealed the growth of bacteria after 24 h of incubation 37 °C and was indicated by growth of green-colored metallic-sheen colonies (Supplementary Fig. S3A). All *E*. *coli* isolates were confirmed positive to cherry color red formation after adding Kovac’s reagent which gave positive result of indole test (Supplementary Fig. S3B). Based on the Gram staining reaction, all the presumptive *Salmonella* and *E*. *coli* isolates were Gram negative and rod-shaped (Supplementary Table S1; Supplementary Fig. S4).

All the isolates of *E*. *coli* were confirmed negative for oxidase and urease tests with no purple colour formation. *E. coli* isolates were positive to MR test and a bright red coloration was produced. Supplementary Table S1 shows the biochemical reactions done on the isolates and the identities of the isolates. Based on morpho-cultural characteristics and biochemical tests, 18 and 24 bacterial isolates were identified as *Salmonella* spp. and *E. coli*, respectively.

### Enumeration of total bacterial load/count in retail fish

**Fig. 1:**
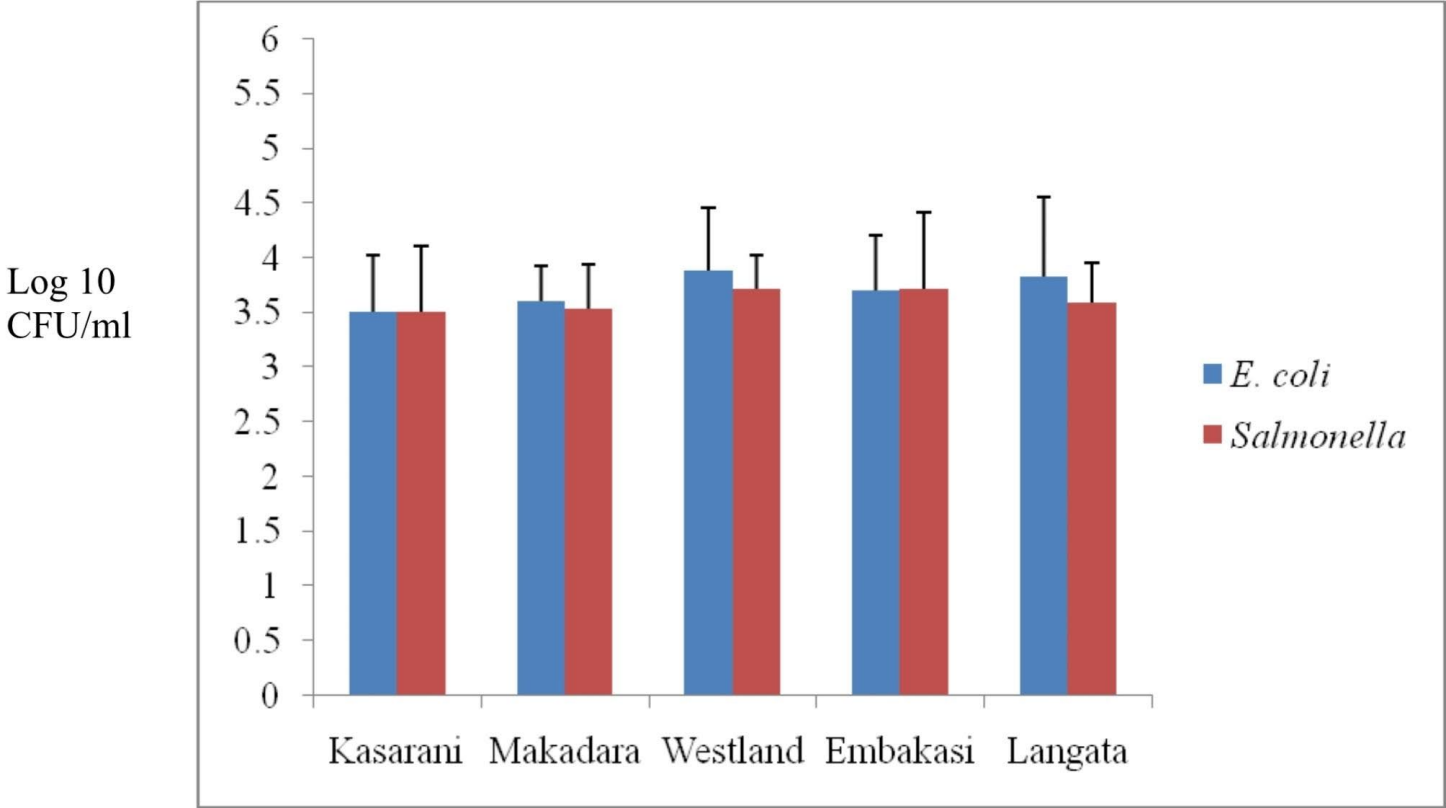
*Salmonella* spp. and *E. coli* counts in fish from five locations of Nairobi County, Kenya. No significant difference (*p* = 0.192) was observed in both *Salmonella* spp. and *E. coli* counts in the five sampling locations

*Salmonella* spp. counts ranged from 3.50 Log CFU/ml to 3.72 Log CFU/ml while *Escherichia coli* counts ranged from 3.51 Log CFU/ml to 3.88 Log CFU/ml across the different markets from five sampled locations (Fig. 1). There was no significant difference (*p* = 0.192) in the *Salmonella* spp. and *E. coli* counts in the five sampling locations.

### Occurrence of *Salmonella* spp. and *E. coli* in retail Nile tilapia samples

**Fig. 2:**
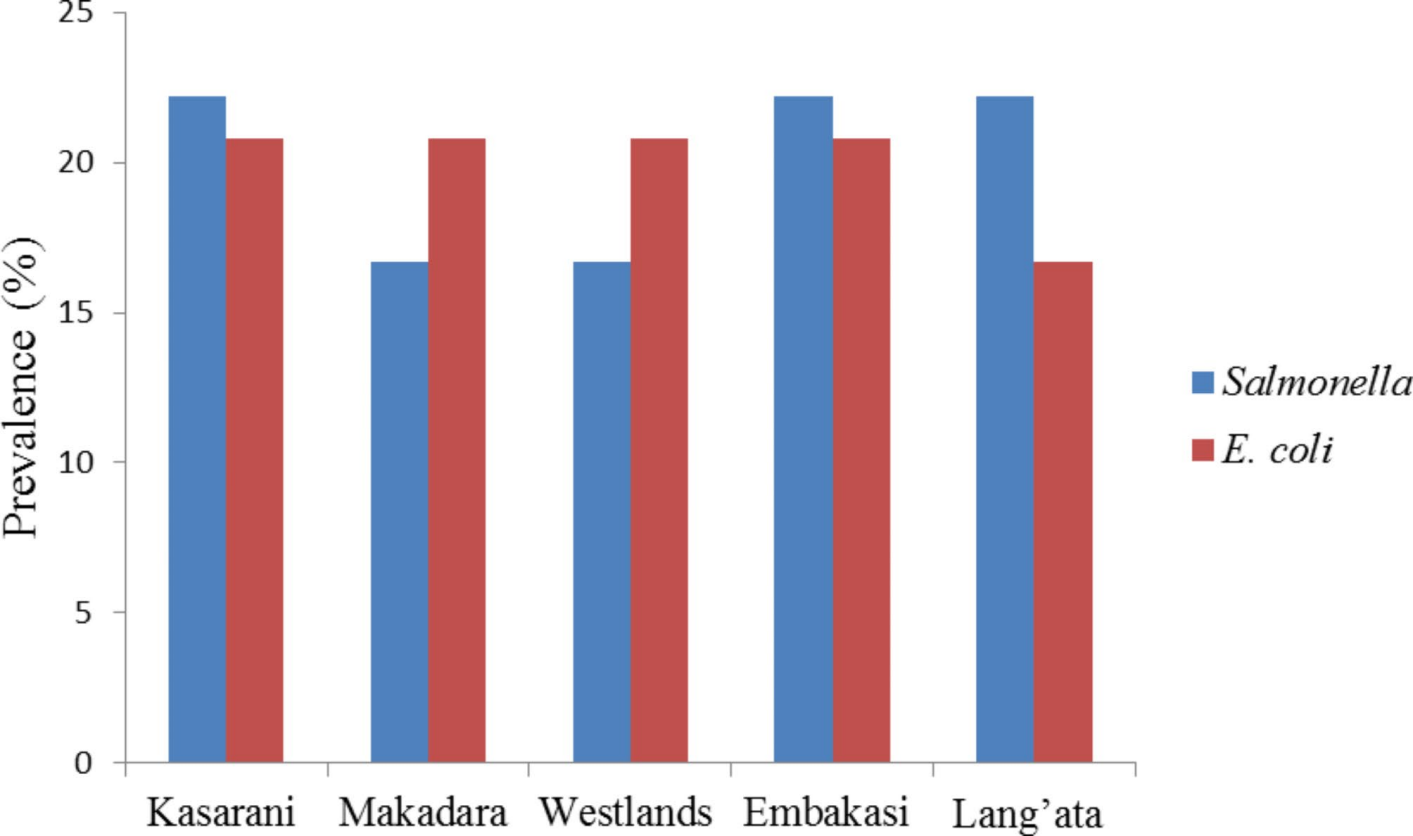
Prevalence of *Salmonella* spp. and *E. coli* isolated from Nile tilapia collected from five locations in Nairobi, Kenya. For *Salmonella* isolates, n = 4, 3, 3, 4, and 4 for Kasarani, Makadara, Westlands, Embakasi and Lang’ata, respectively. For *E. coli*, n = 5, 5, 5, 5 and 4 for Kasarani, Makadara, Westlands, Embakasi and Lang’ata, respectively

The occurrence and contamination rates of *Salmonella* spp. and *E. coli* ranged from 16.7 to 22.2% and 16.7–20.8%, respectively (Fig. 2). The number of *Salmonella* spp. in retail markets within each of the five sampling locations were 4 (22.2%), 3 (16.7%), 3 (16.7%), 4 (22.2%), and 4 (22.2%), for Kasarani, Makadara, Westlands, Embakasi and Lang’ata, respectively. For *E. coli*, the number of isolates were 5 (20.8%) for Kasarani, Makadara, Westlands and Embakasi and 4 (16.7%) for Lang’ata. Overall, the prevalence of contamination of Nile tilapia fish with *Salmonella* spp. and *E. coli* was 26.47% (18/68) and 35.29% (24/68), respectively.

### Occurrence of antimicrobial resistant *Salmonella* species and *E. coli* isolates

The distribution of antimicrobial susceptibility profile of *Salmonella* spp. and *E. coli* is presented in Table 2. *Salmonella* spp. isolates showed resistance to all the antibiotics tested except to cefepime. The highest resistance of *Salmonella* spp. isolates from samples collected from all the locations were to penicillin (22.2%) followed by vancomycin (16.7%). Resistance to ampicillin, cefpodoxime and rifampicin was 11.1%, whereas resistance to ceftazidime, chloramphenicol, meropenem, nitrofurantoin and streptomycin was 5.5%. None of the *Salmonella* spp. isolates was resistant to cefepime. Intermediates were common in all the antibiotics tested with the range of 11.1–61.1% (Table 2).

**Table 2:** Antibiotic resistance patterns of *Salmonella* spp. and *E. coli* isolates

Sub-County/Location	Microorganisms & Isolate ID	Phenotype*													
	*Salmonella* spp.														
		AX	CPM	CPD	CAZ	C	MRP	NIT	P	RIF	S	VA	Resistance (%)	Intermediate (%)	Susceptible (%)
Makadara	MAK-22	R	S	S	I	S	I	R	R	R	S	R	45.5	18.2	36.4
	MAK-01	S	S	S	I	S	I	S	S	S	S	S	0	18.2	81.8
	MAK-02	I	S	S	I	S	I	S	I	I	S	I	0	54.5	45.5
Embakasi	EMB-32	R	S	S	R	R	R	I	S	I	I	R	45.5	27.3	27.3
	EMB-02	I	S	S	S	I	S	S	S	I	S	S	0	27.3	72.7
	EMB-03	S	S	S	I	S	I	I	I	I	I	I	0	63.6	36.4
	EMB-07	S	S	I	S	I	S	I	S	S	I	S	0	36.4	63.6
Langata	LAN-16	S	S	S	I	S	S	S	I	I	S	S	0	27.3	72.7
	LAN-28	I	I	S	S	S	I	I	R	S	I	I	9.1	54.5	36.4
	LAN-15	I	S	I	I	S	S	I	I	S	I	I	0	63.6	36.4
	LAN-20	S	S	I	I	S	I	I	R	S	S	S	9.1	36.4	54.5
Westlands	WES-09	I	S	R	S	S	S	I	S	R	R	R	36.4	18.2	45.5
	WES-01	S	S	S	I	S	I	I	S	S	S	S	0	27.3	72.7
	WES-02	I	S	S	I	S	I	S	I	I	I	I	9.1	63.6	36.4
Kasarani	KAS-01	S	I	R	S	S	S	S	I	I	S	I	9.1	36.4	54.5
	KAS-05	S	S	I	I	S	S	I	R	S	I	S	9.1	36.4	54.5
	KAS-06	S	S	S	I	S	S	S	S	I	S	S	0	18.2	81.8
	KAS-07	I	S	S	S	I	S	I	I	I	S	I	0	54.5	45.5
Resistance (%)		11.1	0	11.1	5.5	5.5	5.5	5.5	22.2	11.1	5.5	16.7			
Intermediate (%)		38.9	11.1	22.2	61.1	16.7	44.4	55.6	38.9	50	38.9	38.9			
Susceptible (%)		50	88.9	66.7	33.3	77.8	50	38.9	38.9	38.9	55.6	44.4			
	***Escherichia coli***														
**Sub-County/Location**		**AX**	**CPM**	**CPD**	**CAZ**	**C**	**MRP**	**NIT**	**P**	**RIF**	**S**	**VA**	**Resistance (%)**	**Intermediate (%)**	**Susceptible (%)**
Makadara	MAK-26	R	S	S	S	S	R	S	S	R	S	R	36.4	0	63.6
	MAK-01	I	S	S	S	S	I	S	I	I	I	I	0	54.5	45.5
	MAK-12	I	S	I	S	S	I	S	S	I	I	R	9.1	45.5	45.5
	MAK-13	I	S	S	S	S	I	S	R	I	S	I	9.1	36.4	54.5
	MAK-15	S	I	I	S	S	I	S	S	R	S	I	9.1	36.4	54.5
Embakasi	EMB-01	S	I	I	S	S	R	S	I	I	S	S	9.1	36.4	54.5
	EMB-12	I	I	I	S	S	I	S	S	S	I	I	0	54.5	45.5
	EMB-11	S	S	I	I	S	I	S	I	I	S	S	0	45.5	54.5
	EMB-10	I	S	R	S	S	I	S	I	S	S	S	9.1	27.3	63.6
	EMB-15	S	S	S	S	S	I	S	S	S	S	S	0	9.1	90.9
Langata	LAN-35	R	S	S	I	S	R	S	S	R	S	R	36.4	9.1	54.5
	LAN-20	I	I	S	I	S	S	S	R	S	S	I	9.1	36.4	54.5
	LAN-23	I	I	I	S	S	I	S	R	S	I	R	18.2	45.5	36.4
	LAN-24	I	I	R	S	S	S	S	I	S	I	I	9.1	45.5	45.5
Westlands	WES-01	I	S	S	I	S	S	S	R	I	I	S	9.1	36.4	54.5
	WES-02	S	S	I	S	R	I	S	S	S	I	I	9.1	36.4	54.5
	WES-03	I	S	I	R	S	I	S	I	I	I	I	9.1	63.6	27.3
	WES-04	I	I	I	S	S	I	S	I	S	S	I	0	54.5	45.5
	WES-05	I	S	S	S	S	I	S	R	S	S	S	9.1	18.2	72.7
Kasarani	KAS-11	I	S	I	S	S	S	S	I	R	S	S	9.1	27.3	63.6
	KAS-12	S	S	I	S	S	S	S	I	S	I	S	0	27.3	72.7
	KAS-13	S	S	S	S	S	I	S	I	S	S	S	0	18.2	81.8
	KAS-14	S	I	S	S	S	S	S	I	R	I	I	9.1	36.4	54.5
	KAS-15	S	S	S	I	S	S	S	S	R	I	S	9.1	18.2	72.7
Resistance (%)		8.3	0	8.3	4.2	4.2	12.5	0	20.8	25	0	16.7			
Intermediate (%)		54.2	33.3	45.8	20.8	0	58.3	0	45.8	29.2	45.8	41.7			
Susceptible (%)		37.5	66.7	45.8	75	95.8	29.2	100	33.3	45.8	54.2	41.7			

AX = Ampicillin/Cloxacillin; CPM = Cefepime; CPD = Cefpodoxime; CAZ = Ceftazidime; C = Chloramphenicol; MRP = Meropenem; NIT = Nitrofurantoin; P = Penicillin-G; RIF = Rifampicin; S = Streptomycin;VA = Vancomycin. The classes of antibiotics are: Carbapenems (MRP), Cephalosporin (third generation; CAZ), Penicillin (P-G), Beta-lactam (AX), Cephalosporin (third generation; CPD), Phenicol (C), Cephalosporin (fourth generation, CPM), Nitrofurans (NIT), Glycopeptides (VA), Ansamycin (RIF), and Aminoglycosides (S)

The results are depicted by: susceptible (S), Intermediate (I), or resistant (R) to each antibiotic

The percentage of resistance to the antibiotics differed among the *E. coli* isolates, of which 25% were resistant to rifampicin. Resistant to penicillin-G, vancomycin, and meropenem was 20.8%, 16.7% and 12.5%, respectively (Table 2). None of the *E. coli* isolates were resistant to cefepime, nitrofurantoin and streptomycin (Table 2). A higher percentage of intermediate isolates 58.3% was observed in meropenem (Table 2). For both *Salmonella* spp. and *E. coli* isolates, the new generation cephalosporins such as cefepime (fourth generation) were completely effective against all the isolates.

### Multidrug resistant patterns of the *S. typhimurium* and *E. coli* isolates

The different *Salmonella* spp. and *E. coli* isolates exhibited a diverse pattern of resistance to a minimum of one class and a maximum of 5 classes of antimicrobials, among the 9 classes tested. The distribution of MDR and MAR index of *S. typhimurium* and *E. coli* isolates is presented in Table 3. Isolates were classified as multi-drug resistant (MDR) if they were resistant to at least three different classes of antibiotics. Based on this classification, 5 isolates for both *S*. *typhimurium* and *E. coli* were MDR. In this study we found that 3 out of 18 (16.7%) *S*. *typhimurium* isolates from fresh Nile tilapia fish were resistant to at least three different classes of antibiotics and were considered to be MDR isolates (Table 3). The three *S*. *typhimurium* isolates were resistant to five antibiotics (AX + NIT + P + RIF + VA and AX + CAZ + C + MRP + VA) which belonged to 5 different classes of antibiotics with a MAR index of 0.45. Two *E. coli* isolates were resistant to five antibiotics (AX + MRP + RIF + VA) which belonged to four different classes of antibiotics with a MAR index of 0.36 (Table 3).

**Table 3:** Distribution of multiple antibiotic resistant characterizations of the *Salmonella* spp. and *E. coli* isolated from fresh Nile tilapia sold in retail markets in Nairobi

Microorganism	Antibiotic resistant pattern	No. of antibiotics (Classes)	No. of resistant species (%)	MDR pattern	MAR index
*Salmonella* spp. (n = 18)					
KAS-01	CPD	1 (1)	1 (5.6)	No	0.09
WES-02	CAZ	1 (1)	1 (5.6)	No	0.09
KAS-05, LAN-20, LAN-28	P	1 (1)	3 (16.7)	No	0.09
WES-09	CPD + RIF + S + VA	4 (4)	1 (5.6)	Yes	0.36
MAK-22	AX + NIT + P + RIF + VA	5 (5)	1 (5.6)	Yes	0.45
EMB-32	AX + CAZ + C + MRP + VA	5 (5)	1 (5.6)	Yes	0.45
*E. coli* (n = 24)					
WES-02	C	1 (1)	1 (4.2)	No	0.09
MAK-12	VA	1 (1)	1 (4.2)	No	0.09
EMBA-01	MRP	1 (1)	1 (4.2)	No	0.09
MAK-13, LAN-20, WES-01, WES-05	P	1 (1)	4 (16.7)	No	0.09
MAK-15, KAS-11, KAS-14, KAS-15	RIF	1 (1)	4 (16.7)	No	0.09
LAN-23	P + VA	2 (2)	1 (4.2)	No	0.18
MAK-26, LAN-35	AX + MRP + RIF + VA	4 (4)	2 (8.3)	Yes	0.36

AX = Ampicillin/Cloxacillin; CPM = Cefepime; CPD = Cefpodoxime; CAZ = Ceftazidime; C = Chloramphenicol; MRP = Meropenem; NIT = Nitrofurantoin; P = Penicillin-G; RIF = Rifampicin; S = Streptomycin; VA = Vancomycin. MAR = Multiple antibiotic resistance. The classes of antibiotics are: Carbapenems (MRP), Cephalosporin (third generation; CAZ), Penicillin (P-G), Beta-lactam (AX), Cephalosporin (third generation; CPD), Phenicol (C), Cephalosporin (fourth generation, CPM), Nitrofurans (NIT), Glycopeptides (VA), Ansamycin (RIF), and Aminoglycosides (S)

### ESBL production in MDR *Salmonella* spp. and *E. coli* isolates

All MDR *Salmonella* spp. and *E. coli* isolates were ESBL producers (Table 4), as determined by the Modified Double Disc Synergy Test method.

**Table 4:** Resistance and ESBL production test results of MDR *Salmonella* spp. and *E. coli* isolates

Microorganism	Isolate ID code	Antibiotic resistance	ESBL production
*Salmonella* spp.			
	WES-09	CPD + RIF + S + VA	Positive
	MAK-22	AX + NIT + P + RIF + VA	Positive
	EMB-32	AX + CAZ + C + MRP + VA	Positive
*E. coli*			
	MAK-26	AX + MRP + RIF + VA	Positive
	LAN-35	AX + MRP + RIF + VA	Positive

AX = Ampicillin/Cloxacillin; CPD = Cefpodoxime; CAZ = Ceftazidime; C = Chloramphenicol; MRP = Meropenem; NIT = Nitrofurantoin; P = Penicillin-G; RIF = Rifampicin; S = Streptomycin; VA = Vancomycin. The classes of antibiotics are: Carbapenems (MRP), Cephalosporin (third generation; CAZ), Penicillin (P-G), Beta-lactam (AX), Cephalosporin (third generation; CPD), Phenicol (C), Nitrofurans (NIT), Glycopeptides (VA), Ansamycin (RIF), and Aminoglycosides (S). ESBL, Extended-spectrum β-lactamase. Positive means that the bacteria produce extended-spectrum β-lactamases, which make them resistant to beta-lactamase antibiotics.

### Molecular identification of MDR *Salmonella* spp. and *E. coli* isolates

For the molecular identification of the recovered MDR isolates, DNA were extracted and 1500 bp size of 16S rRNA genes were amplified and sequenced. The sequences of 16S rRNA gene of five MDR isolates were deposited in the NCBI database under accession numbers OP293362.1, OP293363.1 and OP293364.1 for *S*. *typhimurium* and OP293365.1 and OP293366.1 for *Escherichia coli*. BLASTn results revealed that the three isolates WES-09, MAK-22 and EMB-32 were closely related to *S*. *typhimurium*NR_074910.1 with similarity of 93%, 91% and 93%. The two isolates MAK-26 and LAN-35 were closely related to *E*. *coli* NR_114042.1 with identity percentage of 90% and 93%, respectively (Supplementary Table S2).

### 16S rRNA phylogenetic analysis

**Fig. 3:**
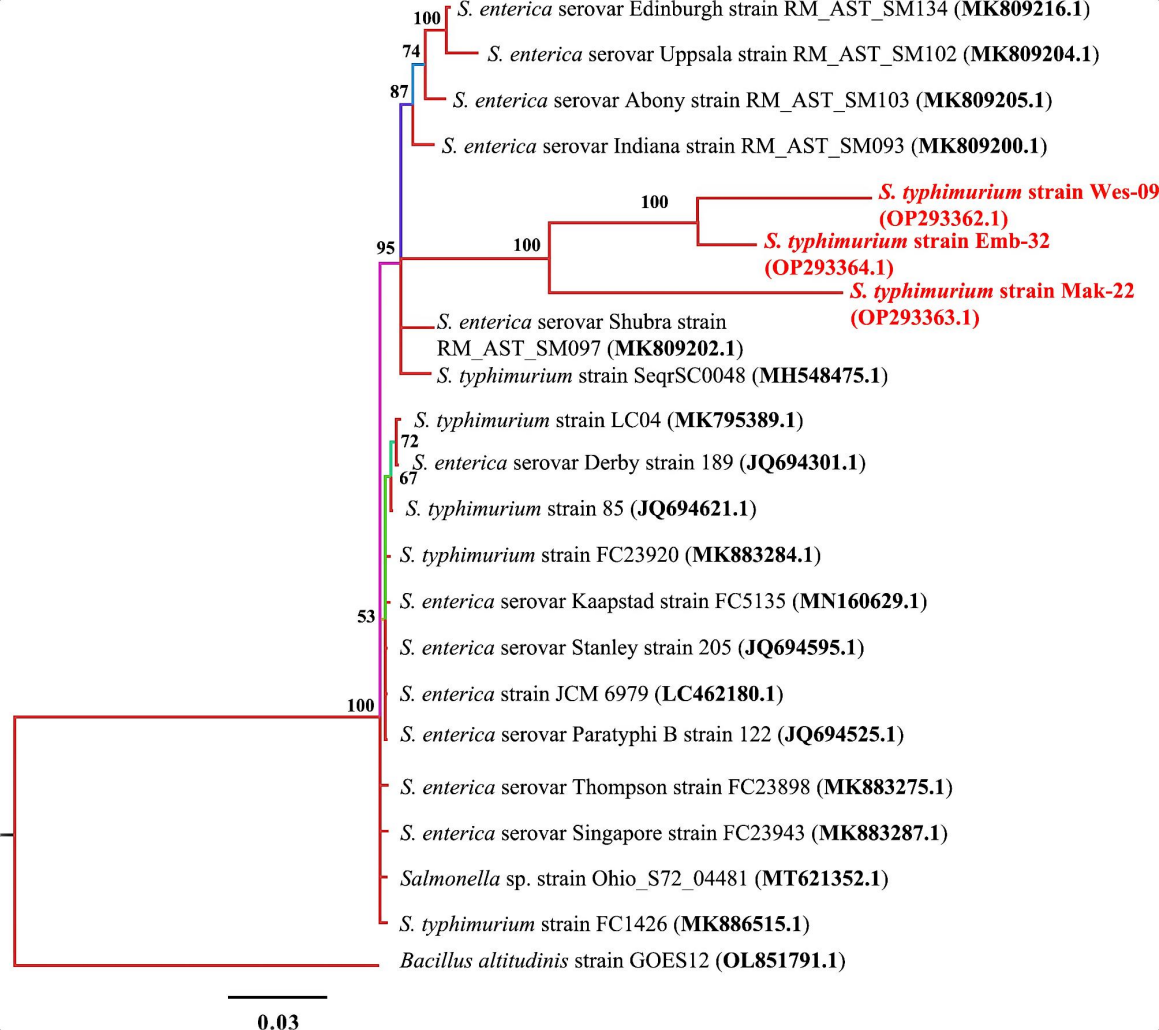
Phylogenetic tree built using eighteen16S rRNA sequences of *Salmonella* spp. New isolates *S. typhimurium* strains WES-09, MAK-22 and EMB-31 are shown in red. Numbers indicated on the nodes are percent posterior probabilities showing statistical support for each node. Branches are coloured based on percent posterior probabilities. The scale bar below the tree indicates the number of expected changes (or substitutions) per site. The outgroup *Bacillus altitudinis* strain GOES12 (OL851791.1) was used in rooting the tree

The phylogenetic analysis (Fig. 3) of 16S rRNA sequence of the three MDR *Salmonella* spp. isolates (OP293362.1, OP293363.1 and OP293364.1) showed distinct clustering and had the same node showing that they both evolved from the same ancestor. The three isolates clustered together in the same cladograph and had 100% homology.

**Fig. 4:**
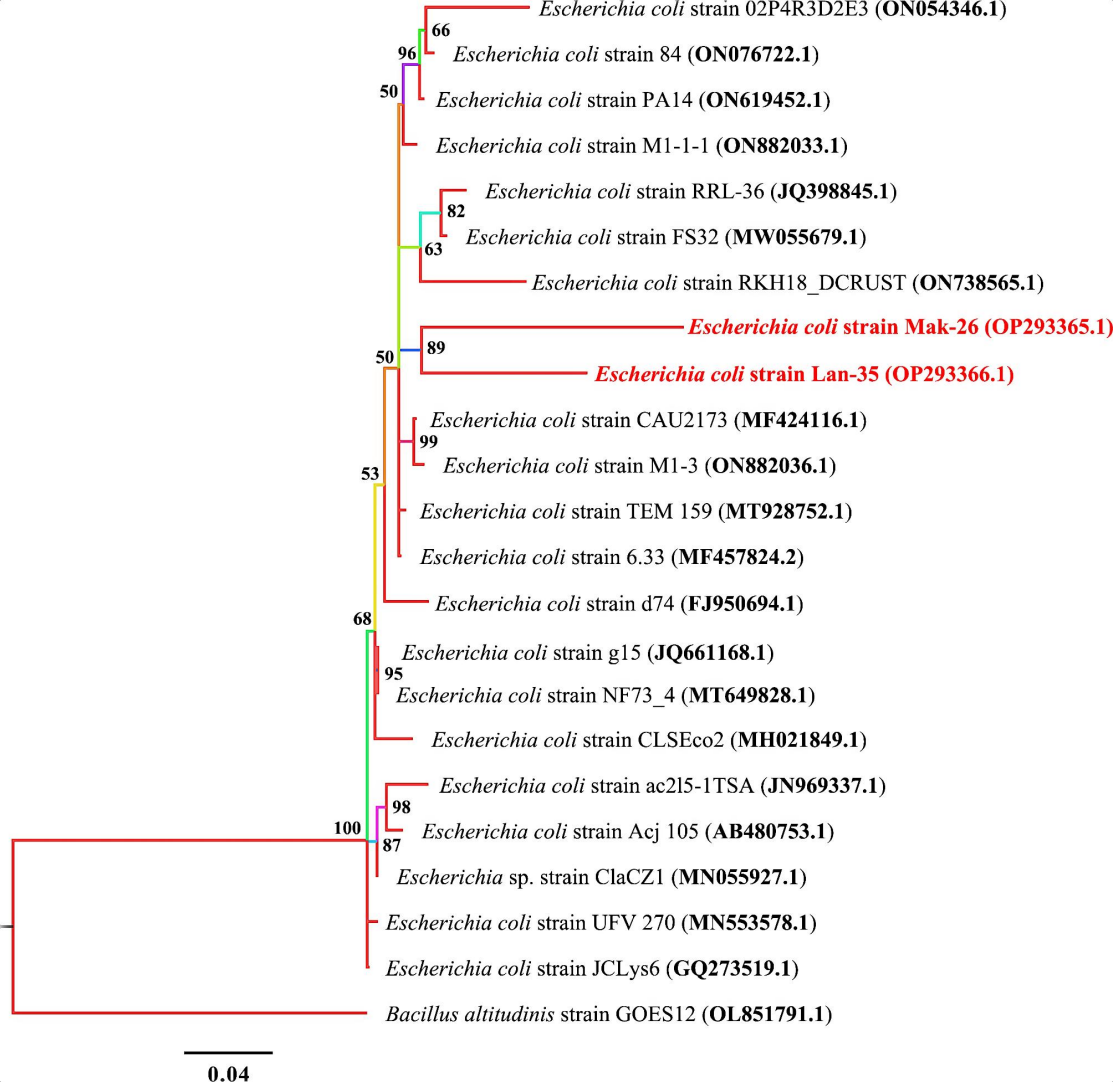
Phylogenetic treebuilt using 16S rRNA sequences of *Escherichia coli* species. New isolates of *E. coli* strains MAK-26 and LAN-35 are shown in red. Numbers indicated on the nodes are percent posterior probabilities showing statistical support for each node. Branches are coloured based on percent posterior probabilities. The scale bar below the tree indicates the number of expected changes (or substitutions) per site. The outgroup *Bacillus altitudinis* strain GOES12 (OL851791.1) was used in rooting the tree

The phylogenetic analysis (Fig. 4) of 16S rRNA sequence of the two MDR *E. coli* isolates (OP293365.1 and OP293366.1) showed distinct clustering and had the same node showing that they both evolved from the same ancestor.

### Detection of virulence-associated genes

**Fig. 5:**
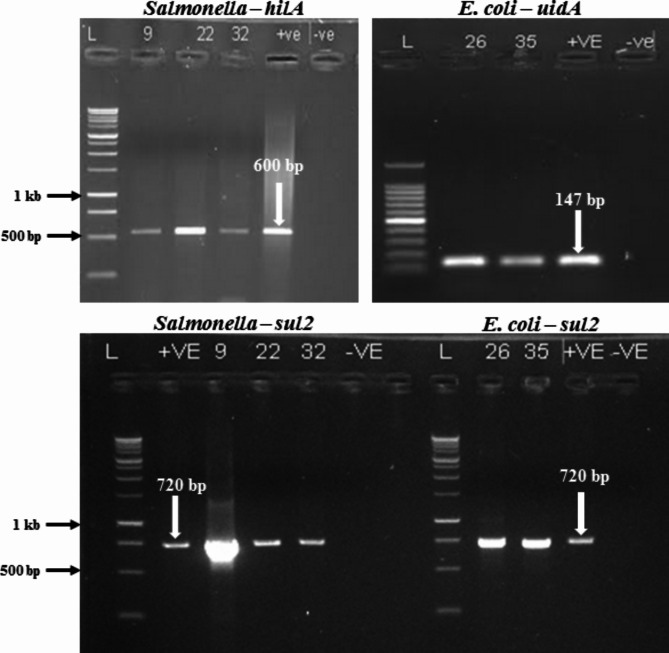
Agarose gel image showing virulence-associated genes *hilA* and *uidA* for *S*. *typhimurium* and *E. coli*, respectively, and antibiotic resistance gene (*sul2*) for *S*. *typhimurium* and *E. coli*. Lane L: Molecular weight marker, Lane + ve: positive control, Lane -ve: negative control, Lanes 9, 22 and 32: MDR *Salmonella* spp. isolates, Lanes 26 and 35: MDR *E. coli* isolates

The distribution of virulence genes among the multidrug resistant (MDR) *S*. *typhimurium* and *E. coli* are presented in Table 5. All the three MDR *S*. *typhimurium* harbored *invA* (*Salmonella* invasion gene) and *hilA*, whereas the two MDR *E*. *coli* isolates contained *uidA* (Fig. 5).

**Table 5:** Multidrug resistance patterns, virulence genes, and drug resistance-associated genes of MDR *S. typhimurium* and *E*. *coli* isolates

Pathogens	Antibiotic resistant pattern	No. of antibiotics (Classes*)	Virulence genes			Antibiotic resistant associated genes										
			*invA*	*hilA*	*uidA*	*tetA*	*tetC*	^*bla*^TEM-1	^*bla*^CMY-2	^*bla*^CTX-M	^*bla*^Z	*sul2*	*catI*	*dfrA7*	*strA*	*aadA*
*S. typhymurium* WES-09	CPD, RIF, S, VA	4 (4)	+	+	NT		+	+	+	+	+	+		+	+	+
*S. typhymurium* MAK-22	AX, NIT, P-G, RIF, VA	5 (5)	+	+	NT	+	+	+	+	+	+	+		+	+	+
*S. typhymurium* EMB-32	AX, CAZ, C, MRP, VA	5 (5)	+	+	NT	+	+	+	+	+	+	+	+		+	+
*E. coli* MAK-26	AX, MRP, RIF, VA	4 (4)	NT	NT	+	+	+	+	+	+	+	+		+	+	+
*E. coli* LAN-35	AX, MRP, RIF, VA	4 (4)	NT	NT	+		+	+	+	+	+	+		+	+	+

AX = Ampicillin/Cloxacillin; CPM = Cefepime; CPD = Cefpodoxime; CAZ = Ceftazidime; C = Chloramphenicol; MRP = Meropenem; NIT = Nitrofurantoin; P = Penicillin-G; RIF = Rifampicin; S = Streptomycin; VA = Vancomycin. NT = Not Tested

TetA and tetC = Tetracycline resistant genes, ^*bla*^TEM-1, ^*bla*^CMY-2, ^*bla*^CTX-M, ^*bla*^Z = Beta lactamases-encoding genes, *catI* = chloramphenicol resistant gene, *sul2* = sulphonamide resistant gene, *dfrA7* = Trimethoprim resistant gene, *strA* = streptomycin inactivating gene and *aadA* = Aminoglycoside resistant genes

+ indicates the presence of the resistance genes following amplification by PCR; − indicates the absence of the target resistance genes following amplification by PCR

*Classes of antibiotics are: Carbapenems (MRP), Cephalosporin (third generation; CAZ), Penicillin (P-G), Beta-lactam (AX), Cephalosporin (third generation; CPD), Phenicol (C), Cephalosporin (fourth generation, CPM), Nitrofurans (NIT), Glycopeptides (VA), Ansamycin (RIF), and Aminoglycosides (S)

### Detection of antimicrobial resistance genes

The distribution of antibiotic-resistant elements among the multidrug resistant (MDR) *S*. *typhimurium* and *E. coli* are presented in Table 5. Among the *S*. *typhimurium* screened for resistant genes ^*bla*^TEM-1, ^*bla*^CMY-2, ^*bla*^CTX-M, and ^*bla*^Z (beta-lactamase resistance genes), *catI* (chloramphenicol resistant genes) in EMB-32 only, *sul*2 (sulphonamide resistant gene), *str*A (streptomycin inactivating gene), *aad*A (aminoglycoside resistant gene) and *tet*C (tetracycline resistance gene) were present. No amplification of *tet*A and *dfr*A7 was observed in *S*. *typhimurium* WES-09 and *S*. *typhimurium* EMB-32 isolates, respectively.

The two *E. coli* isolates tested positive for antimicrobial resistant genes ^*bla*^TEM-1, ^*bla*^CMY-2, ^bla^CTX-M, ^*bla*^Z (beta–lactamase resistance gene), *sul*2 (sulphonamide resistant gene), *str*A (streptomycin inactivating gene), *aad*A, *tet*C (tetracycline resistance gene) and *dfr*A7. The strain *E. coli* (LAN-35) showed no amplification for *tet*A gene. Figure 5 shows a representative agarose gel of the amplification of tested antibiotic resistant genes in MDR *S*. *typhimurium* and *E. coli*. The presence of beta-lactamase genes was detected in all tested isolates (Table 5), confirming the phenotypic results of ESBL production tests.

## Discussion

*Salmonella* spp. and pathogenic *E. coli* are the most frequent causes of foodborne illnesses worldwide and deaths due to consumption of contaminated food and food products. In the present study, *Salmonella* spp. and *E*. *coli* were isolated from 26.47% to 35.29% of the raw Nile tilapia fish samples, respectively. The prevalence of *Salmonella* spp. in this study was lower than that reported by Budiati et al. [22] in tilapia samples from Malaysia. Also our findings inconsistent with Lerma-Fierro et al. [53] who detected *Salmonella* spp. in 41.7% of fresh Nile tilapia fillets marketed in Tepic Nayarit City, Mexico. Higher prevalence of *Salmonella* spp. of 64.9% and 75% were also reported previously in tilapia imported from Thailand [54] and fresh tilapia samples collected from different markets in Bangladesh [55], respectively. Various countries have different hygienic control and management programs, which may explain the differences in *Salmonella* spp. contamination rates of Nile tilapia fish. Another reason, for these differences may be due to sample size and sample types. *Salmonella* spp. and pathogenic *E*. *coli* are found in animal or human reservoir and their presence in raw Nile tilapia fish from retail markets in this study suggests poor hygienic practices during production, handling, processing and marketing, which could be as a result of direct or indirect fecal contamination, posing risk to people consuming raw or undercooked contaminated fish.

Foodborne pathogens transmitted from fish, fish products and seafood products can lead to serious infections or even death. It is estimated that more than 12% of food-borne outbreaks related to the consumption of fish are caused by bacteria pathogens [56, 57].Total bacteria count in fish is an important parameter in assessing the level of contamination, qualityand public health concern. Several indicator bacteria are not pathogenic themselves but their abundance represents potential risks of contamination [58]. In this study, high counts of *Salmonella* spp. and *E. coli* were obtained from the fish samples collected from all the five different locations/sites in Nairobi County, and this confirms previous reports that *Salmonella* spp. and *E. coli* are important foodborne pathogens of animal-derived foods. The findings from this study are similar to the high total bacterial counts in fish observed in other studies [58, 59].The presence of *Salmonella* spp. and *E. coli* in fish is due to the fact that aquatic environment is tremendously vulnerable to pollution and run-off from anthropogenic sources which contaminate fish products representing a potential hazard to humans [60]. *E. coli* is one of the major pathogenic microorganisms that may reach animal-derived foods and is an indication of faecal contamination from warm-blooded animals [61]. *E. coli* are commensal bacteria and *E. coli* pathotypes can cause zoonotic disease that poses a public health risk. The presence of *E. coli* in thefish samples sold in open-air markets could be due to poor handling of fish by traders as well as unhygienic handling during transportation and storage. The useof contaminated water for cleaning and processing of fish in the markets mayalso contribute to the secondary contamination. The lack of proper drainage facilities and heavy flyinfestation in these fish markets also promotes tertiary contamination to a great extent [59].

The examination of *Salmonella* spp. and *E. coli* isolated from fish for resistance to 11 antibiotics from 9 different classes of antibiotics revealed the existence of antibiotic resistant phenotypes. The isolated *Salmonella* spp. and *E. coli* showed resistance to ampicillin/Cloxacillin, ceftazidime, chloramphenicol, nitrofurantoin, rifampicin, streptomycin, penicillin-G, rifampicin, cefpodoxime, meropenem and vancomycin which is in agreement with a study by Sifuna et al. [62]. This antibiotic resistant profile can be due to the frequent use of antibiotics in fish for therapeutic and growth promotion [63, 64]. The presence of antibiotic-resistant *Salmonella* spp. and *E. coli* in fresh Nile tilapia fish indicates the role of these fish as spreaders of resistant microorganisms in aquatic environments. The low resistance observed in chloramphenicol by *Salmonella* spp. and *E. coli* could be as a result of its ban on usage, since it inhibits protein translation causing aplastic anaemia in some patients [65]. Of specific concern is the high rate of resistance seen to streptomycin, as this is one of the watch group antibiotics in the WHO Access, Watch and Reserve (AWaRe) classification of antibiotics for the evaluation and monitoring of use [66]. Resistance to carbapenems (meropenem) may be due to the transmission of bacteria from human sources, especially that carbapenems are not approved for use in food-animals [67]. According to the WHO, carbapenem-resistant *Salmonella* spp. and *E. coli* are considered to be among the most critical pathogens [68]. The detection of carbapenem-resistant *Salmonella* spp. and *E. coli* in fish has to be treated as an urgent public health problem. Vancomycin is an antibiotic of the last resort in bacterial infections [69], so presence of vancomycin resistant *Salmonella* spp. and *E. coli* isolates in this study is a concern for consumer health.

The morphological characteristics observed in this study support the previous observation contained in the WHO Global Salm-Surv as described by Henderiksen et al. [70] for *Salmonella* spp. and also in the manual for identification of medical bacteria as described by Phillips [71] for *E. coli*. The 16S rRNA sequencing further confirmed the *Salmonella* spp. and *E. coli* isolates, and clustered them into closely related phylogenetic clades. The 16S rRNA gene is an important landmark in the study of the evolution and classification of bacteria, and has served as base molecular identification tool for study of evolutionary relationships among groups of bacteria [72]. Based on the results from this study, all the MDR *Salmonella* spp. and *E. coli* were identified as *S*. *typhymurium* WES-09, *S. typhymurium* MAK-22, *S*. *typhimurium* EMB-32 and *E*. *coli* MAK-26 and *E*. *coli* LAN-35. PCR is a robust and rapid detection method with increased sensitivity and specificity for detecting *Salmonella* spp. in food, environmental, and clinical samples [73]. Detection of virulence factors is a key step to identify the potential pathogenicity of the obtained bacteria isolates. Fish surface and tissue invasion by the bacterial pathogens is considered to be facilitated by the functioning of virulence factors [74]. The *invA* gene has been the target for many PCR protocols, as it is found in almost all known serovars of *Salmonella* spp. [75]. This gene encodes an inner membrane protein necessary for invasion of epithelial cells by *Salmonella* spp. [76]. The occurrence of the MDR invasive *Salmonella* spp. isolates among the fish samples suggests that consumers and other stakeholders within the food and value chain might be at a risk of *Salmonella*-borne infections.

In this study, we report the detection of MDR *S*. *typhimurium* and *E. coli* from Nile tilapia fish marketed for human consumption. The presence of MDR isolates from fresh Nile tilapia fish investigated indicate that consumers are exposed to disease-causing pathogens that make treatment challenging. This is significant to human health due to the zoonotic nature of these pathogens. To avoid the development of MDR, the use of antibiotics should be more strategic and selective. Given that fish harbour multiple bacterial communities living in close proximity to each other, antibiotic resistance in some of these bacteria could lead to easy transfer of resistant genes to others. This could result in increased spread of antibiotic resistance to humans. The spread of MDR bacteria could also be exacerbated by consumption of raw, undercooked or insufficiently heat-treatedfish and fish products. Based on these results, we propose improving sanitary handling and processing of fish to reduce the risk of spread of bacteria pathogens capable of spreading antibiotic resistant genes to humans. Our study highlight the serious issue of *S*. *typhimurium* and *E. coli* multidrug resistance in retail Nile tilapia fish which could result in the evolution of *S*. *typhimurium* and *E. coli* into a super bacteria and risk to public health.

The emergence and spread of ESBL *Salmonella* spp. and *E. coli* have become a public health concern because of their association with morbidity and mortality and reduced treatment options. Our results showed that all the MDR *S*. *typhimurium* isolates showed resistance to antibiotic classes important in human medicine such as beta-lactamases. Therefore, we conducted a double-disc synergy test (DDST) for ESBL phenotype production and our experiments indicated that all the MDR isolates had an ESBL phenotype [77]. The detection of beta-lactamase genes using PCR was performed to confirm the phenotypic pattern. The beta-lactamase genes ^*bla*^TEM-1, ^*bla*^CMY-2, ^*bla*^CTX–M, and ^*bla*^Z were present in all the MDR *S*. *typhimurium* and *E. coli* isolates. The ^*bla*^CTX-M genes encode for ESBLs frequently identified in Gram-negative pathogens. These types of enzymes are active against cephalosporins and monobactams (but not cephamycins or carbapenems), and are of great epidemiological and clinical interest [78].

In the current study, the MDR *S*. *typhimurium* and *E. coli* isolates were examined by PCR to identify the antibiotic resistant genes. PCR analysis revealed the presence of antibiotic-resistance genes belonging to β-lactamases, tetracycline-resistant, sulfonamide-resistant, trimethoprim-resistant and aminoglycosides-resistant genes. Genes like *aadA*, *dfrA7* and *sul2* detected in MDR *S. typhimurium* and *E. coli* often co-exist as part of gene cassettes on class 1 integrons [79]. The class 1 resistance integrons is located on mobile elements like transposons and plasmids and is widely distributed among clinical and environmental isolates and plays an important role as reservoirs of antimicrobial resistance genes [80, 81]. Amplification of ^*bla*^TEM-1, ^*bla*^CMY-2, ^*bla*^CTX-M, and ^*bla*^Z is attributed to long term exposure of β-lactam antibiotics in animal and fish farming and for treatment of Gram-negative infections [82]. The presence of antibiotic resistant genes shared across the bacterial isolates reflects active horizontal gene transfer among bacteria in aquaculture. Horizontal gene transfer (HGT) allows bacteria to exchange their genetic materials including antibiotic resistance genes (ARGs) among different species [83], hence promoting multidrug resistance. The use of antimicrobial agents in aquaculture for long periods of time have contributed to increase of antibacterial resistance in fish pathogens, emergence of antimicrobial resistant bacteria in aquatic environments, and also increasing the potential to transfer these resistant genes to pathogenic bacteria of terrestrial animals and humans [84]. The use of antibiotics in aquatic culture in Kenya is not regulated and their indiscriminate use has led to the rise of antibiotic resistant bacteria hence the transfer of the resistance to human bacteria. Therefore there is need for studies to understand the epidemiology of antibiotics in aquaculture in Kenya, as this will be an important step in solving the problem of antibiotic resistance in the aquaculture environment.

## Conclusions

The isolation and characterization of *S*. *typhimurium* and *E. coli*, especially those with multiple antimicrobial resistance, in Nile tilapia in retail markets is of public health concern. The occurrence of multidrug-resistant isolates is of specific concern for human and domestic animal health.The potential ability of these MDR bacteria to enter into the food chain can expose humans to serious health risks. This calls for application of more hygienic practices during all stages of fish production and processing for selling. Further, it requires a wide microbiological surveillance and strict governing of the uncontrolled use of antimicrobials either for treatment or as growth promoters, not only in fish production but also in other livestock production systems. Till date, there is insufficient data regarding the role of wildlife and the environment in the complex mechanism of antimicrobial resistance. Therefore, there is a need for research on antibiotic susceptibility surveillance in aquatic environments where fresh fish and marine fish are obtained for human consumption.

### Electronic supplementary material

Below is the link to the electronic supplementary material.

**Supplementary Table 1** Morphological characteristics, Gram staining and biochemical tests of bacteria isolates obtained from raw Nile tilapia fish sold in retail markets for human consumption, Nairobi County, Kenya. **Supplementary Table 2** Similarity of 16S rRNA sequences of antibiotic resistant *E*. *coli* and *S*. *typhimurium* isolates from Nile tilapia, compared with accessions from the GenBank database. **Supplementary Figure 1***Salmonella* spp. on XLD showing black centered colonies. **Supplementary Figure 2***Salmonella* spp., on TSI showing red slant, yellow butt, H_2_S and gas (cracks in the medium). **Supplementary Figure 3** (A) *E. coli* showing blue-green metallic sheen color colonies on Eosin Methylene Blue agar, (B) Indole positive of *E. coli* with cherry red ring formation. **Supplementary Figure 4** (A) Gram staining of *E. coli*; (B) Gram staining of *Salmonella* spp.

## Data Availability

The datasets generated and/or analyzed during the current study are available from the corresponding author on reasonable request. The 16S rRNAsequences obtained in this study were deposited in Genebank through online submission portal under accession numbers OP293362.1, OP293363.1 and OP293364.1 for *S*. *typhimurium* and OP293365.1 and OP293366.1 for *E*. *coli*.

## References

[1] Acar J, Davies J, Buckley M (2009). Antibiotic resistance: an ecological perspective on an old problem.

[2] WHO. National Action Plan on Prevention and Containment of Antimicrobial Resistance, 2017–2022. Regional Office for Africa: Brazzaville, Republic of Congo,; 2020.

[3] Ferri M, Ranucci E, Romagnoli P, Giaccone V (2017). Antimicrobial Resistance: A Global emerging threat to Public Health systems. Crit Rev Food Sci Nutr.

[4] Lulijwa R, Rupia EJ, Alfaro AC (2020). Antibiotic use in aquaculture, policies and regulation, health and environmental risks: a review of the top 15 major producers. Rev Aqua.

[5] DewiRR, Hassan L, Daud HM, Matori MF, NordinF, Ahmad NI, Zakaria Z. Prevalence and Antimicrobial Resistance of *Escherichia coli*, *Salmonella* and *Vibrio* Derived from Farm-Raised Red Hybrid Tilapia (*Oreochromis* spp.) and Asian Sea Bass (Lates Calcarifer, Bloch 1970) on the West Coast of Peninsular Malaysia. Antibiotics. 2022;11:136. 10.3390/antibiotics11020136 PMC8868497 35203739

[6] Gauthier DT (2015). Bacterial zoonoses of fishes: a review and appraisal of evidence for linkages between fish and human Infections. Vet J.

[7] Anderson JL, Asche F, Garlock T, Chu J (2017). Aquaculture: its role in the future of food. Front Econ Glob.

[8] Food and Agriculture Organization (FAO) (2017). The state of World fisheries and Aquaculture.

[9] Obwanga B, Soma K, Ingasia AO, Rurangwa E, Wonderen D, van Beekman G, Kilelu C. Exploring enabling factors for commercializing the aquaculture sector in Kenya. *Research Report* 011, Wageningen University & Research, Wageningen. 2020.

[10] Adeleke B, Robertson-Andersson D, Moodley G, Taylor S (2020). Aqua-culture in Africa: a comparative review of Egypt, Nigeria, and Uganda vis-a-vis South Africa. Rev Fish Sci Aquac.

[11] Shrestha A, Chaudhary CK, Ghale R, Shrestha A (2018). Monosex male tilapia production. Int J Anim Biotechnol Appl.

[12] Obiero KO, Kyule D, Opiyo MA, Abwao J, Kirimi JG, Outa N, Muthoka M, Githukia CM, Ogello EO (2022). Nile tilapia (*Oreochromis niloticus* Linnaeus, 1758) culture in Kenya: emerging production technologies and socio-economic impacts on local livelihoods. Aquaculture Fish and Fisheries.

[13] Kromhout D, Bosschieter EB, de Lezenne CC (1985). The inverse relation between fish consumption and 20-year mortality from coronary Heart Disease. N Engl J Med.

[14] Bashir I, Lone FA, Bhat RA, Mir SA, Dar ZA, Dar SA, Hakeem K, Bhat R, Qadri H, editors. Concerns and threats of contamination on aquatic ecosystems. In Bioremediation and Biotechnology; *Springer*: Berlin/Heidelberg, Germany, 2020;1–26.

[15] Patel M, Kumar R, Kishor K, Mlsna T, Pittman CU, Mohan D (2019). Pharmaceuticals of emerging concern in aquatic systems: Chemistry, occurrence, effects, and removal methods. Chem Rev.

[16] Smith P (2008). Antimicrobial resistance in aquaculture. Rev Sci Technol off Int Epizoot.

[17] Herrera FC, Santos JA, Otero A, García-López ML (2006). Occurrence of foodborne pathogenic bacteria in retail prepackaged portions of marine fish in Spain. J Appl Microbiol.

[18] Onyuka JHO, Kakai R, Onyango DM, Arama PF, Gichuki J, Ayub V (2011). Prevalence and Antimicrobial Susceptibility Patterns of Enteric Bacteria Isolated from Water and Fish in Lake Victoria Basin of Western Kenya. World Acad Sci Eng and Technol.

[19] Abebe E, Gugsa G, Ahmed M. Review on major food-borne zoonotic bacterial pathogens. J Trop Med. 2020. 4674235. 10.1155/2020/4674235 PMC7341400 32684938

[20] Wang M, Lu M. Tilapia polyculture: a global review. Aquac Res 2015;1–12.

[21] Fernandes DVGS, Castro VS, da Cunha Neto A, Figueiredo EES (2018). *Salmonella* spp. in the fish production chain: a review. Cienc Rural.

[22] Budiati T, Rusul G, Wan-Abdullah WN, Arip YM, Ahmad R, Thong KL (2013). Prevalence, antibiotic resistance and plasmid profiling of *Salmonella* in catfish (*Clarias gariepinus*) and tilapia (*Tilapia mossambica*) obtained from wet markets and ponds in Malaysia. Aquaculture.

[23] Le HV, Kawahara R, Khong DT, Tran HT, Nguyen TN, Pham KN, Jinnai M, Kumeda Y, Nakayama T, Ueda S (2015). Widespread dissemination of extended-spectrum-lactamase-producing, multidrug-resistant *Escherichia coli* in livestock and fishery products in Vietnam. Int J Food Contam.

[24] Bali EB, Acik L, Sultan N (2010). Phenotypic and molecular characterization of SHV, spectrum beta-lactamase (CTX-M-3 like) from India and gene association with insertion sequence ISEcp1. Microbiol Lett.

[25] Hassuna NA, Khairalla AS, Farahat EM, Hammad AM, Abdel-Fattah M (2020). Molecular characterization of extended-spectrum β-lactamase-producing *E. Coli* recovered from community-acquired urinary tract Infections in Upper Egypt. Sci Rep.

[26] Schwaber MJ, Navon-venezia S, Schwartz D, Schwaber MJ, Navon-venezia S, Schwartz D (2005). High levels of antimicrobial coresistance among extended-spectrum-beta-lactamase-producing Enterobacteriaceae. Antimicrob Agents Chemother.

[27] Chandel DS, Johnson JA, Chaudhry R, Sharma N, Shinkre N, Parida S et al. Extended-spectrum β-lactamase-producing gram-negative bacteria causing neonatal sepsis in India in rural and urban settings. J Med Microbiol. 2011:500–7. 10.1099/jmm.0.027375-0 PMC3133666 21183602

[28] Yu ZN, Wang J, Ho H, Wang YT, Huang SN, Han RW (2020). Prevalence and antimicrobial-resistance phenotypes and genotypes of *Escherichia coli* isolated from raw milk samples from mastitis cases in four regions of China. J Glob Antimicrob Resist.

[29] Cheesbrough M (1985). Medical laboratory manual for tropical countries. Microbiol Eng Lang Book Soc Lond.

[30] Slaby BM, Martin RE, Ramsdell GE (1981). Reproducibility of microbiological counts on frozen cod: a collaborative study. J Food Sci.

[31] Collins CH, Lyne MP. Microbiological methods, fifth ed. Butterworth, London. UK.1984.

[32] Mohamed SMN, Walaa AH, Wafaa MKB (2019). Evaluation of antibiotic susceptibility test results: how guilty a laboratory could be?. J Egypt Pub Health Assoc.

[33] Standards OIEOIE. Guidelines and resolution on Antimicrobial Resistance and the Use of Antimicrobial agents. World Organisation for Animal Health; 2015.

[34] WHO. WHO List of Critically Important Antimicrobials (CIA) 2018.

[35] CLSI M100. Performance Standards for Antimicrobial Susceptibility Testing an Informational Supplement for Global Application Developed through the Clinical and Laboratory Standards Institute Consensus Process. CLSI: Malvern, PA, USA., 2021, 27.

[36] Krumperman PH (1983). Multiple antibiotic resistance indexing of Escherichia coli to identify high-risk sources of fecal contamination of foods. Appl Environ Microbiol.

[37] Wose KCN, Ateba N, Kawadza TD (2010). Antibiotic resistance profiles of *Esch*e*richia coli* isolated from different water sources in the Mmabatho locality, North-West Province, South Africa. Res Lett.

[38] Kaur J, Shashi C, Sheevani GM (2013). Modified double disc synergy test to detect ESBL production in urinary isolates of *Escherichia coli* and *Klebsiella pneumoniae*. J Clin Diagn Res.

[39] Hall TA, BioEdit. A user-friendly biological sequence alignment editor and analysis program for Windows 95/98/NT. Nucleic Acids Symposium Series, 1999;41:95–98.

[40] Edgar RC. MUSCLE: a multiple sequence alignment method with reduced time and space complexity. BMC Bioinformatics, 2004;5(1). 10.1186/1471-2105-5-113 PMC517706 15318951

[41] Fontana C, Favaro M, Pelliccioni M, Pistoia ES, Favalli C (2005). Use of the MicroSeq 16S rRNA gene-based sequencing for identification of bacterial isolates that commercial automated systems failed to identify correctly. J Clin Microbiol.

[42] Bhatta DR, Bangtrakulnonth A, Tishyadhigama P, Saroj SD, Bandekar JR, Hendrikesn RS, Kapadnis BP, Serotyping (2007). PCR, phage-typing and antimicrobial sensitivity testing of *Salmonella* serovars isolated from urban drinking water supply systems of Nepal. Lett Appl Microbiol.

[43] Nora CC, Eliana RP, Margarita CO (2002). Detection of *hil*A gene sequences in serovars of *Salmonella enterica* sub species Enterica. Mem Inst Oswaldo Cruz.

[44] Tsai LY, Palmer CL, Sangeermano LR (1993). Detection of *Escherichia coli* in sludge by polymerase chain reaction. Appl Environ Microbiol.

[45] Fang H, Ataker F, Hedin G, Dornbusch K (2008). Molecular epidemiology of extended-spectrum beta-lactamases among Escherichia coli isolates collected in a Swedish hospital and its associated health care facilities from 2001 to 2006. J Clin Microbiol.

[46] Edelstein M, Pimkin M, Palagin I, Palagin I, Edelstein I, Stratchonski L. Prevalence and molecular epidemiology of CTX-M extended spectrum beta-lactamase-producing *Escherichia coli* and *Klebsiella pneumoniae* in Russian hospitals. Antimicrob. Agents Chemother. 2003;47(12):3724–32. 10.1128/AAC.47.12.3724-3732.2003 PMC296190 14638473

[47] Baddour MM, Abuelkheir MM, Fantana AJ (2007). Comparison of mecA polymerase chain reaction with phenotypic methods for the detection of methicillin-resistant *Staphyloccocus aureus*. Curr Microbiol.

[48] Maynard C, Bekal S, Sanschagrin F, Levesque RC, Brousseau R, Masson L, Lariviere S, Harel J (2004). Heterogeneity among virulence and antimicrobial resistance gene profiles of extraintestinal *Escherichia coli* isolates of animal and human origin. J Clin Microbiol.

[49] Ribeiro VB, Lincopan N, Landgraf M, Franco BDGM, Destro MT (2011). Characterization of class 1 integrons and antibiotic resistance genes in multidrug-resistant Salmonella enterica isolates from foodstuff and related sources. Brazilian J Microbiol.

[50] Ng LK, Martin I, Alfa M, Mulvey M (2001). Multiplex PCR for the detection of tetracycline resistant genes. Mol Cell Probes.

[51] Grape M, Motakefi A, Pavuluri S, Kahlmeter G (2007). Standard and real-time multiplex PCR methods for detection of trimethoprim resistance dfr genes in large collections of bacteria. Clin Microbiol Infect.

[52] Velusamy S, Barbara EG, Mark JL, Lien TN, Susan IH, Ynte HS, Stephen PO (2007). Phenotypic and genotypic antimicrobial resistance patterns of *Escherichia coli* isolated from dairy cows with mastitis. Vet Microbiol.

[53] Lerma-Fierro AG, Flores-Lo´pez MK, Guzma´n-Robles ML, Corte´s-Sa´nchez AJ (2020). Microbiological evaluation of minimally processed and marketed fish in popular market of the city of Tepic Nayarit, Mexico sanitary quality of tilapia (*Oreochromis niloticus*). Tropic.

[54] Elhadi N (2014). Prevalence and antimicrobial resistance of *Salmonella* spp. in raw retail frozen imported freshwater fish to Eastern Province of Saudi Arabia. Asian Pac J Trop Biomed.

[55] Seel SK, Kabir SML, Islam MA (2016). Molecular detection and characterization of *Salmonella* spp. isolated from fresh fishes sold in selected Upazila markets of Bangladesh. Bangl J Vet Med.

[56] Huss H, Jørgensen LV, Vogel BF (2000). Control options for listeria monocytogenes in seafoods. Int J Food Microbiol.

[57] Aberoumand A (2010). Estimation of microbiological variations in minced lean fish products. World J Fish and Marine Sci.

[58] Nabeel MA, Zenon BB, Haitham AA, Mohammed AMA, Abdulaziz MA (2017). Culture-depended bacteria in commercial fishes: qualitative assessment and molecular identification using 16S rRNA gene sequencing. Saudi J Biol Sci.

[59] Marijani E (2022). Prevalence and antimicrobial resistance of Bacteria isolated from Marine and Freshwater Fish in Tanzania. Int J Microbiol.

[60] Sichewo PR, Gono RK, Muzvondiwa JV, Sizanobuhle N (2013). Isolation and identification of pathogenic bacteria in edible fish: a case study of fletcher dam. Int J Sci Res.

[61] Chao KK, Chao CC, Chao WL (2003). Suitability of the traditional microbial indicators and their enumerating methods in the assessment of fecal pollution of subtropical freshwater environments. J Microbiol Immunol Infect.

[62] Sifuna AW, Njagi ENM, Okemo P, Munyalo A, Orinda GO, Kariuki S (2008). Microbological quality and safety of *Restrineobola Argentea* retailed in Kisumu town markets, Kenya. East Afri Med J.

[63] Alderman D, Hastings TS (1998). Antibiotic use in aquaculture: development of antibiotic resistance–potential for consumer health risks. Int J Food Sci Technol.

[64] Cabello FC (2006). Heavy use of prophylactic antibiotics in aquaculture: a growing problem for human and animal health and for the environment. Environ Microbiol.

[65] Berendsen B, Stolker L, de Jong J, Nielen M, Tserendorj E, Sodnomdarjaa R, Cannavan A, Elliott C (2010). Evidence of natural occurrence of the banned antibiotic chloramphenicol in herbs and grass. Anal Bioanal Chem.

[66] World Health Organization. WHO Access, Watch, Reserve (AWaRe) Classification of Antibiotics for Evaluation and Monitoring of Use. 2021. (WHO/MHP/HPS/EML/2021.04); WHO: Geneva, Switzerland, 2021.

[67] Poirel L, Stephan R, Perreten V, Nordmann P (2014). The carbapenemase threat in the animal world: the wrong culprit. J Antimicrob Chemother.

[68] Tacconelli E, Carrara E, Savoldi A, Harbarth S, Mendelson M, Monnet DL, Pulcini C, Kahlmeter G, Kluytmans J, Carmeli Y (2018). Discovery, research, and development of new antibiotics: the WHO priority list of antibiotic-resistant bacteria and Tuberculosis. Lancet Infect Dis.

[69] Boneca IG, Chiosis G (2003). Vancomycin resistance: occurrence, mechanisms and strategies to combat it. Expert Opin Ther Targets.

[70] Hendriksen R, Jaap W, Van Bergen M. Global Salm-Surv. A Global S*almonella* Surveillance and Laboratory Support Project of the World Health Organization. Identification of Thermotolerant Campylobacter. WHO;; 2003.

[71] Phillips I, Cowan (1993). Steels. Manual for the identification of medical bacteria. J Clin Pathol.

[72] Hoque MN, Istiaq A, Clement RA, Sultana M, Crandall KA, Siddiki AZ, Hossain MA (2019). Metagenomic deep sequencing reveals association of microbiome signature with functional biases in bovine mastitis. Sci Rep.

[73] Toze S (1999). PCR and the detection of microbial pathogens in water and wastewater. Water Res.

[74] Sen K, Lye D (2007). Importance of flagella and enterotoxins for *Aeromonas* virulence in a mouse model. Can J Microbiol.

[75] Chiu CH, Ou JT (1996). Rapid identification of *Salmonella* serovars in feaces by specific detection of virulence genes, invA and spvC, by an enrichment broth culture-multiplex PCR combination assay. J Clin Microbiol.

[76] Darwin KH, Miller VL (1999). Molecular basis of the Interaction of Salmonella with the intestinal mucosa. Clin Microbiol Rev.

[77] European Food Safety Authority; European Centre for Disease Prevention and Control (2021). The European Union Summary Report on Antimicrobial Resistance in Zoonotic and Indicator bacteria from humans, animals and food in 2018/2019. EFSA J.

[78] Cantón R, Coque TM (2006). The CTX-M β-Lactamase pandemic. Curr Opin Microbiol.

[79] Sung JY, Koo SH, Kwon KC (2014). Epidemiological characterizations of class 1 integrons from multidrug-resistant *Acinetobacter* isolates in Daejeon, Korea. Ann Lab Med.

[80] Koczura R, Mokracka J, Barczak A, Krysiak N, Kubek M, Kaznowski A (2013). Association between the presence of class 1 integrons virulence genes and phylogenetic groups of *Escherichia coli* isolates from river water. Microb Ecol.

[81] Koczura R, Semkowska A, Mokracka J (2014). Integron-bearing Gram-negative bacteria in lake waters. Lett Appl Microbiol.

[82] Zaniani FR, Meshkat Z, Nasab MN (2012). The prevalence of TEMand SHV genes among extended-spectrum beta-lactamases producing *Escherichia coli* and *Klebsiella pneumoniae*. Iran J Basic Med Sci.

[83] Le Roux F, Blokesch M (2018). Eco-evolutionary dynamics linked to horizontal gene transfer in *Vibrios*. Annu Rev Microbiol.

[84] Miller RA, Harbottle H. Antimicrobial drug resistance in fish pathogens. Microbiol. Spectrum. 2017;6(1): ARBA-0017-2017. 10.1128/microbiolspec.arba-0017-2017 PMC11633554 29372680

