# Supplementary material for: Antimicrobial resistance profiles of salmonella spp. and escherichia coli isolated from fresh nile tilapia (oreochromis niloticus) fish marketed for human consumption
Source: BMC Microbiol. 2023 Oct 26;23:306. doi: 10.1186/s12866-023-03049-8 (PMC10601154; doi:10.1186/s12866-023-03049-8)
Supplement: Supplementary file 1 — Supplementary Table 1 Morphological characteristics, Gram staining and biochemical tests of bacteria isolates obtained from raw Nile tilapia fish sold in retail markets for human consumption, Nairobi County, Kenya. Supplementary Table 2 Similarity of 16S rRNA sequences of antibiotic resistant E. coli and S. typhimurium isolates from Nile tilapia, compared with accessions from the GenBank database. Supplementary Figure 1Salmonella spp. on XLD showing black centered colonies. Supplementary Figure 2Salmonella spp., on TSI showing red slant, yellow butt, H2S and gas (cracks in the medium). Supplementary Figure 3 (A) E. coli showing blue-green metallic sheen color colonies on Eosin Methylene Blue agar, (B) Indole positive of E. coli with cherry red ring formation. Supplementary Figure 4 (A) Gram staining of E. coli; (B) Gram staining of Salmonella spp. [file 12866_2023_3049_MOESM1_ESM.doc]

**Supplementary Material**

Antimicrobial resistance profiles of *Salmonella* and *Escherichia coli* isolated from fresh Nile tilapia (*Oreochromis niloticus*) fish marketed for human consumption

Millicent T. Mumbo^1,2,3^, Evans N. Nyaboga^1*^, Johnson K. Kinyua^2^, Edward K. Muge^1^, Scholastica G. K. Mathenge^3^, Henry Rotich^4^, Geoffrey Muriira^4^, Bernard Njiraini^4^, and Joshua M. Njiru^4^

^1^Department of Biochemistry, University of Nairobi, Nairobi, Kenya

^2^Department of Biochemistry, Jomo Kenyatta University of Agriculture and Technology (JKUAT), Nairobi, Kenya

^3^Department of Medical Laboratory Science, Kenyatta University, Nairobi, Kenya

^4^Research and development Department, Kenya Bureau of Standards, Nairobi, Kenya

*Corresponding author:

Dr. Evans N. Nyaboga, PhD

Department of Biochemistry, University of Nairobi, Nairobi, Kenya

Email: [nyaboga@uonbi.ac.ke](mailto:nyaboga@uonbi.ac.ke)

**Supplementary Tables**

**Supplementary Table 1** Morphological characteristics, Gram staining and biochemical tests of bacteria isolates obtained from raw Nile tilapia fish sold in retail markets for human consumption, Nairobi County, Kenya.

| **Strain** | **Colour** | **Shape** | **Size (mm)** | **Texture** | **Opacity** | **Margin** | **Elevation** | **TSI** | | | | **CT** | **OX** | **I** | **CI** | **UR** | **MR-VP** | | **Gram**  **stain** | **Cell**  **Shape** | **Possible identity** |
| --- | --- | --- | --- | --- | --- | --- | --- | --- | --- | --- | --- | --- | --- | --- | --- | --- | --- | --- | --- | --- | --- |
|  |  |  |  |  |  |  |  | **S** | **B** | **H_2_S** | **G** |  |  |  |  |  | **MR** | **VP** |  |  |  |
| MAK-01s | B | Round | 3 | Mucoid | Opaque | Entire | Convex | R | Y | + | + | + | - | NT | + | - | + | - | - | Rod | *Salmonella* spp. |
| WES-01 | B | Round | 2 | Mucoid | Opaque | Entire | Convex | R | Y | + | + | + | - | NT | + | - | + | - | - | Rod | *Salmonella* spp. |
| KAS-01 | B | Round | 2 | Mucoid | Opaque | Entire | Convex | R | Y | + | + | + | - | NT | + | - | + | - | - | Rod | *Salmonella* spp. |
| EMB-01 | G/P | Round | 2.5 | Firm | Opaque | Entire | Convex | nt | nt | - | - | + | - | + | - | - | + | - | - | Rod | *Escherichia coli* |
| WES-01 | G/P | Round | 2 | Firm | Opaque | Entire | Convex | nt | nt | - | - | + | - | + | - | - | + | - | - | Rod | *Escherichia coli* |
| MAK-01e | G/P | Round | 2 | Firm | Opaque | Entire | Convex | nt | nt | - | - | + | - | + | - | - | + | - | - | Rod | *Escherichia coli* |
| MAK-02 | B | Round | 2.5 | Mucoid | Opaque | Entire | Convex | R | Y | + | + | + | - | NT | + | - | + | - | - | Rod | *Salmonella* spp. |
| EMB-02 | B | Round | 3 | Mucoid | Opaque | Entire | Convex | R | Y | + | + | + | - | NT | + | - | + | - | - | Rod | *Salmonella* spp. |
| WES-02s | B | Round | 4 | Mucoid | Opaque | Entire | Convex | R | Y | + | + | + | - | NT | + | - | + | - | - | Rod | *Salmonella* spp. |
| WES-02e | G/P | Round | 2 | Firm | Opaque | Entire | Convex | nt | nt | - | - | + | - | + | - | - | + | - | - | Rod | *Escherichia coli* |
| EMB-03 | B | Round | 3 | Mucoid | Opaque | Entire | Convex | R | Y | + | + | + | - | NT | + | - | + | - | - | Rod | *Salmonella* spp. |
| WES-03 | G/P | Round | 3 | Firm | Opaque | Entire | Convex | nt | nt | - | - | + | - | + | - | - | + | - | - | Rod | *Escherichia coli* |
| WES-04 | G/P | Round | 2 | Firm | Opaque | Entire | Convex | nt | nt | - | - | + | - | + | - | - | + | - | - | Rod | *Escherichia coli* |
| KAS-05 | B | Round | 2 | Mucoid | Opaque | Entire | Convex | R | Y | + | + | + | - | NT | + | - | + | - | - | Rod | *Salmonella* spp. |
| WES-05 | G/P | Round | 2.5 | Firm | Opaque | Entire | Convex | nt | nt | - | - | + | - | + | - | - | + | - | - | Rod | *Escherichia coli* |
| KAS-06 | B | Round | 2.5 | Mucoid | Opaque | Entire | Convex | R | Y | + | + | + | - | NT | + | - | + | - | - | Rod | *Salmonella* spp. |
| EMB-07 | B | Round | 3 | Mucoid | Opaque | Entire | Convex | R | Y | + | + | + | - | NT | + | - | + | - | - | Rod | *Salmonella* spp. |
| KAS-07 | B | Round | 3.5 | Mucoid | Opaque | Entire | Convex | R | Y | + | + | + | - | NT | + | - | + | - | - | Rod | *Salmonella* spp. |
| WES-09 | B | Round | 4 | Mucoid | Opaque | Entire | Convex | R | Y | + | + | + | - | NT | + | - | + | - | - | Rod | *Salmonella* spp. |
| EMB-10 | G/P | Round | 3 | Firm | Opaque | Entire | Convex | nt | nt | - | - | + | - | + | - | - | + | - | - | Rod | *Escherichia coli* |
| EMB-11 | G/P | Round | 3 | Firm | Opaque | Entire | Convex | nt | nt | - | - | + | - | + | - | - | + | - | - | Rod | *Escherichia coli* |
| KAS-11 | G/P | Round | 2 | Firm | Opaque | Entire | Convex | nt | nt | - | - | + | - | + | - | - | + | - | - | Rod | *Escherichia coli* |
| MAK-12 | G/P | Round | 3 | Firm | Opaque | Entire | Convex | nt | nt | - | - | + | - | + | - | - | + | - | - | Rod | *Escherichia coli* |
| EMB-12 | G/P | Round | 2 | Firm | Opaque | Entire | Convex | nt | nt | - | - | + | - | + | - | - | + | - | - | Rod | *Escherichia coli* |
| KAS-12 | G/P | Round | 2.5 | Firm | Opaque | Entire | Convex | nt | nt | - | - | + | - | + | - | - | + | - | - | Rod | *Escherichia coli* |
| MAK-13 | G/P | Round | 3 | Firm | Opaque | Entire | Convex | nt | nt | - | - | + | - | + | - | - | + | - | - | Rod | *Escherichia coli* |
| KAS-13 | G/P | Round | 2 | Firm | Opaque | Entire | Convex | nt | nt | - | - | + | - | + | - | - | + | - | - | Rod | *Escherichia coli* |
| KAS-14 | G/P | Round | 2 | Firm | Opaque | Entire | Convex | nt | nt | - | - | + | - | + | - | - | + | - | - | Rod | *Escherichia coli* |
| LAN-15 | B | Round | 3 | Mucoid | Opaque | Entire | Convex | R | Y | + | + | + | - | NT | + | - | + | - | - | Rod | *Salmonella* spp. |
| MAK-15 | G/P | Round | 2 | Firm | Opaque | Entire | Convex | nt | nt | - | - | + | - | + | - | - | + | - | - | Rod | *Escherichia coli* |
| EMB-15 | G/P | Round | 2 | Firm | Opaque | Entire | Convex | nt | nt | - | - | + | - | + | - | - | + | - | - | Rod | *Escherichia coli* |
| KAS-15 | G/P | Round | 2.5 | Firm | Opaque | Entire | Convex | nt | nt | - | - | + | - | + | - | - | + | - | - | Rod | *Escherichia coli* |
| LAN-16 | B | Round | 4 | Mucoid | Opaque | Entire | Convex | R | Y | + | + | + | - | NT | + | - | + | - | - | Rod | *Salmonella* spp. |
| LAN-20s | B | Round | 4 | Mucoid | Opaque | Entire | Convex | R | Y | + | + | + | - | NT | + | - | + | - | - | Rod | *Salmonella* spp. |
| LAN-20e | G/P | Round | 3 | Firm | Opaque | Entire | Convex | nt | nt | - | - | + | - | + | - | - | + | - | - | Rod | *Escherichia coli* |
| MAK-22 | B | Round | 3.5 | Mucoid | Opaque | Entire | Convex | R | Y | + | + | + | - | NT | + | - | + | - | - | Rod | *Salmonella* spp. |
| LAN-23 | G/P | Round | 3 | Firm | Opaque | Entire | Convex | nt | nt | - | - | + | - | + | - | - | + | - | - | Rod | *Escherichia coli* |
| LAN-24 | G/P | Round | 3 | Firm | Opaque | Entire | Convex | nt | nt | - | - | + | - | + | - | - | + | - | - | Rod | *Escherichia coli* |
| MAK-26 | G/P | Round | 2 | Firm | Opaque | Entire | Convex | nt | nt | - | - | + | - | + | - | - | + | - | - | Rod | *Escherichia coli* |
| LAN-28 | B | Round | 3 | Mucoid | Opaque | Entire | Convex | R | Y | + | + | + | - | NT | + | - | + | - | - | Rod | *Salmonella* spp. |
| EMB-32 | B | Round | 4 | Mucoid | Opaque | Entire | Convex | R | Y | + | + | + | - | NT | + | - | + | - | - | Rod | *Salmonella* spp. |
| LAN-35 | G/P | Round | 2 | Firm | Opaque | Entire | Convex | nt | nt | - | - | + | - | + | - | - | + | - | - | Rod | *Escherichia coli* |

**Key:** B: black centre; G/P: Green metallic sheen on EMB / pink on MacConkey; R: red; Y: yellow; H_2_S: hydrogen sulphide; G: gas; NT: not tested; CT: catalase; OX: oxidase; I: indole test; CI: citrate utilization; UR: urease test; MR: methyl red; VP: Vogues-Proskauer; +: positive; −: negative.

**Supplementary Table 2** Similarity of 16S rRNA sequences of antibiotic resistant *E. coli* and *S.* *typhimurium* isolates from Nile tilapia, compared with accessions from the GenBank database.

| **Isolate ID Code** | **Sub-County/Location** | **16S rRNA Accession** | **Closest Match in Blast** | **Similarity (%)** | **GenBank Accession No.** |
| --- | --- | --- | --- | --- | --- |
| WES-09 | Westland | OP293362.1 | *S. typhimurium* | 93 | NR_074910.1 |
| MAK-22 | Makadara | OP293363.1 | *S. typhimurium* | 91 | NR_074910.1 |
| EMB-32 | Embakasi | OP293364.1 | *S. typhimurium* | 93 | NR_074910.1 |
| MAK-26 | Makadara | OP293365.1 | *E. coli* | 90 | NR_114042.1 |
| LAN-35 | Lang’ata | OP293366.1 | *E. coli* | 93 | NR_114042.1 |

**Supplementary Figures**

*
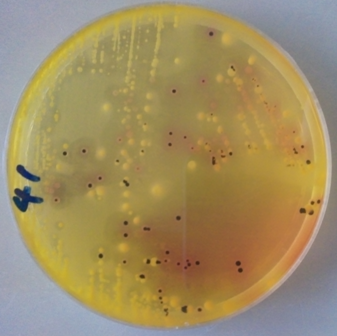
*

**Supplementary Figure 1** *Salmonella* spp. on XLD showing black centered colonies


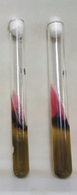

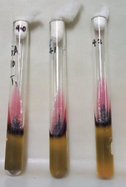


**Supplementary Figure 2** *Salmonella* spp., on TSI showing red slant, yellow butt, H_2_S and gas (cracks in the medium).


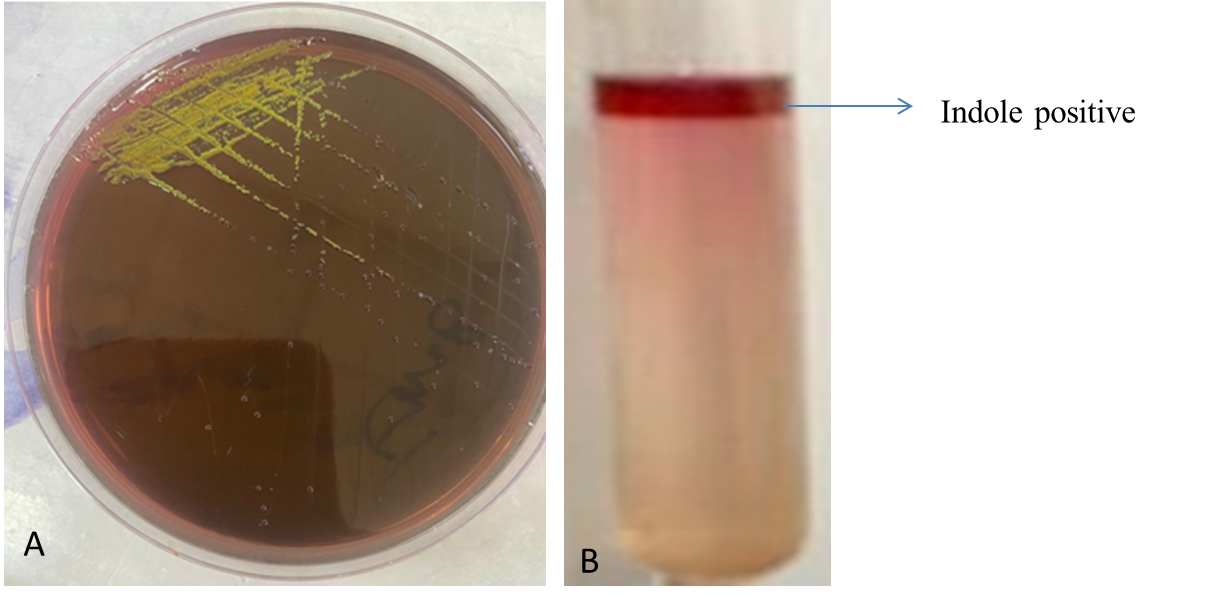


**Supplementary Figure 3** (A) *E*. *coli* showing blue-green metallic sheen color colonies on Eosin Methylene Blue agar, (B) Indole positive of *E*. *coli* with cherry red ring formation


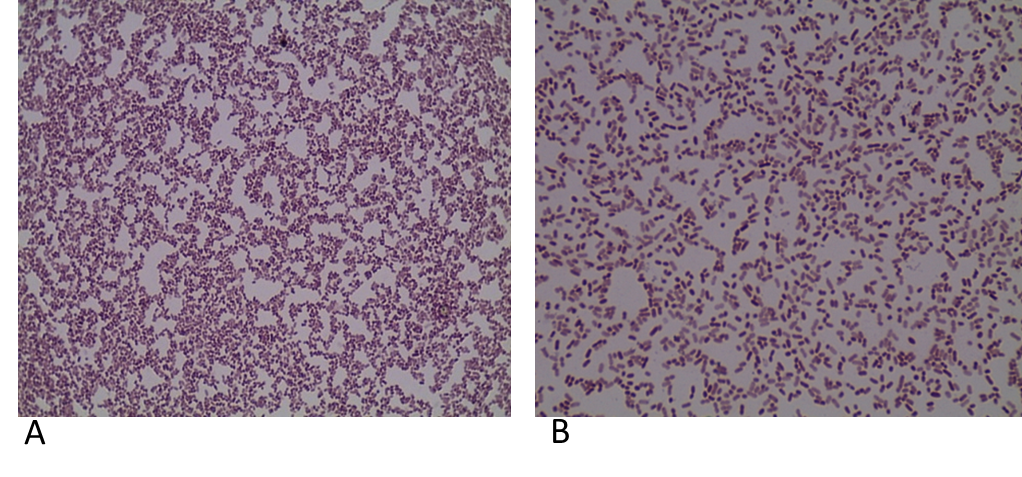


**Supplementary Figure 4** (A) Gram staining of *E. coli*; (B) Gram staining of *Salmonella* spp
